# Prognostic Impact of the Pretreatment Controlling Nutritional Status (CONUT) Score in Anaplastic Thyroid Cancer: A Retrospective Cohort Study

**DOI:** 10.3390/cancers17203344

**Published:** 2025-10-16

**Authors:** Sun-Kyung Park, Nam Kyung Kim, Jun Sung Lee, Hyeok Jun Yun, Yong Sang Lee, Hye Sun Lee, Seok-Mo Kim, Young Song

**Affiliations:** 1 Department of Anesthesiology and Pain Medicine and Anesthesia and Pain Research Institute, Yonsei University College of Medicine, Seoul 03722, Republic of Korea; mayskpark@yuhs.ac; 2 Department of Surgery, Thyroid Cancer Center, Institute of Refractory Thyroid Cancer, Gangnam Severance Hospital, Yonsei University College of Medicine, Seoul 06273, Republic of Korea; nkkim20@yuhs.ac (N.K.K.); LJS-513@yuhs.ac (J.S.L.); gsyhj@yuhs.ac (H.J.Y.); medilys@yuhs.ac (Y.S.L.); 3 Biostatistics Collaboration Unit, Department of Research Affairs, Yonsei University College of Medicine, Seoul 03722, Republic of Korea; hslee1@yuhs.ac

**Keywords:** anaplastic thyroid cancer, malnutrition, Controlling Nutritional Status score, Geriatric Nutritional Risk Index, prognostic factor, Prognostic Nutritional Index

## Abstract

**Background/Objectives**: Anaplastic thyroid cancer (ATC) is an aggressive thyroid cancer subtype with a poor prognosis. The Controlling Nutritional Status (CONUT) score, reflecting both immune and nutritional status, is a prognostic marker in several malignancies; however, its utility in ATC has not been established. We aimed to evaluate the predictive value of the pretreatment CONUT score in ATC and compare its prognostic utility with that of other nutritional indices, including the Prognostic Nutritional Index (PNI) and Geriatric Nutritional Risk Index (GNRI). **Methods**: We retrospectively reviewed clinical characteristics, laboratory parameters, and survival outcomes of 156 patients with ATC at our institution between January 2004 and May 2024. Based on survival analysis, patients were categorized into low- and high-risk groups based on each nutritional index (CONUT score, PNI, GNRI) using optimal cut-off values. One-year survival differences were evaluated using Kaplan–Meier curves and log-rank test. Independent predictors of 1-year mortality were identified using multivariable Cox proportional hazards regression. **Results**: Optimal thresholds were 3, 42, and 102 for the CONUT score, PNI, and GNRI, respectively. Patients with CONUT scores ≥ 3 exhibited significantly higher 1-year mortality, compared with those with scores < 3. Multivariable analysis revealed that CONUT score ≥ 3, PNI ≤ 42, and GNRI ≤ 102 were independently associated with increased 1-year mortality risk. Incorporation of CONUT score ≥ 3 into the baseline prediction model significantly enhanced its discriminatory performance. **Conclusions**: These findings underscore the prognostic value of pretreatment immuno-nutritional assessment and support the integration of the CONUT score into early risk stratification strategies for patients with ATC.

## Simple Summary

Anaplastic thyroid cancer is a rare but highly aggressive malignancy with poor survival outcomes. Simple and reliable markers that can predict prognosis are essential for guiding clinical decision-making. In this retrospective study, we assessed the prognostic value of the Controlling Nutritional Status (CONUT) score, a blood test–based index reflecting both nutritional and immune function. We compared its predictive performance with other commonly used nutritional indices. We found that patients with higher CONUT scores, indicating impaired nutritional and immune status, had a significantly increased risk of 1-year mortality. These findings suggest that incorporating immuno-nutritional assessment, particularly the CONUT score, into routine evaluation may improve early risk stratification and support more personalized approaches in both clinical care and future research for this challenging cancer.

## 1. Introduction

Anaplastic thyroid cancer (ATC) is among the highly lethal types of thyroid malignancy [1,2]. Although ATC represents 1–2% of all thyroid cancers, it is characterized by rapid progression and markedly unfavorable prognosis, with a one-year survival of 20% and a median survival of 3–5 months [1,3,4]. Recent therapeutic advances, including the emergence of immunotherapy and targeted agents, have led to modest improvements in overall survival [1,2,5]. Nonetheless, the aggressive nature of ATC and the need for multidisciplinary care highlight the importance of early prognostic stratification [1,5]. Timely and individualized treatment planning based on risk stratification is essential for improving outcomes [2].

Traditional prognostic assessments for ATC rely primarily on clinicopathological variables such as age, tumor size, TNM stage, and surgical resectability. Although molecular profiling and mutational data have recently improved prognostication and therapeutic decision-making, such analyses are not always feasible in routine practice due to cost, availability, and time constraints [6,7]. This underscores the need for simple, objective, and widely accessible prognostic tools.

In recent years, immuno-nutritional indices have emerged as significant prognostic factors across various malignancies [1,8]. Malnutrition, commonly observed in patients with advanced cancer, has consistently been associated with poor treatment response and reduced survival [9]. To objectively evaluate nutritional and immune status, several scoring systems have been introduced [10,11]. The Controlling Nutritional Status (CONUT) score is commonly used for its simplicity and reliability [8,9,10,12]. This score is derived from serum albumin levels, total cholesterol, and lymphocyte count, thereby reflecting both nutritional reserves and immune competence [8,10]. Recently, the prognostic significance of CONUT has also been reported in thyroid cancer. Dalmiglio et al. found that a higher CONUT score was significantly associated with poorer progression-free survival in patients with advanced thyroid cancer receiving tyrosine kinase inhibitors [9]. Kim et al. showed that higher preoperative CONUT scores were significantly associated with advanced T stage and extrathyroidal extension in patients with papillary thyroid carcinoma [13]. Other indices, such as the Prognostic Nutritional Index (PNI) and Geriatric Nutritional Risk Index (GNRI), have also demonstrated prognostic relevance in several cancers, and PNI has been linked to outcomes in ATC [1]. However, direct comparisons among these indices are limited, and the prognostic role of CONUT in ATC remains inadequately defined.

Therefore, the objective of this study was to evaluate the prognostic utility of the pretreatment CONUT score in patients with ATC. Additionally, we compared its predictive performance for 1-year mortality with that of the PNI and GNRI and identified independent prognostic factors associated with 1-year mortality in this high-risk cohort.

## 2. Materials and Methods

### 2.1. Study Design

This study analyzed the electronic medical record data of 157 patients diagnosed with ATC at our institution. The study was approved by the Institutional Review Board and Hospital Research Ethics Committee of Yonsei University Gangnam Severance Hospital (IRB number: 3-2024-0169; approval date: 26 June 2024). Given the retrospective nature of the study, the Institutional Review Board granted a waiver of informed consent, and this waiver was formally documented as part of the ethics approval. The study was conducted in accordance with the Declaration of Helsinki, as revised in 2013. The study followed the Strengthening the Reporting of Observational Studies in Epidemiology (STROBE) guidelines [14].

### 2.2. Study Population and Treatment Protocol

The study included patients diagnosed with ATC at Gangnam Severance Hospital from January 2004 to May 2024. Eligible patients met the following inclusion criteria: (1) histologically confirmed of ATC based on the fifth edition of the World Health Organization classification of tumors of endocrine organs by surgery or via open biopsy and (2) receiving treatment at our institution. Patients were excluded if clinical data were incomplete or if they were lost to follow-up. All patients received treatment according to the institution’s standardized ATC management protocol, as described in prior publications [3,15]. The detailed treatment protocol is provided in Methods S1 [3,15].

### 2.3. Data Collection and Definitions

Data were obtained from a single-center observational cohort database designed to investigate outcomes in patients with ATC. We extracted patient characteristics such as age, gender, and body mass index (BMI) from the electronic medical records. Additional tumor- and treatment-related variables were also collected: tumor size, the Tumor-Node-Metastasis (TNM) stage, distant metastasis, surgical treatment, type of surgery (excisional biopsy, debulking, or complete resection), chemotherapy and its regimen, radiation therapy, and use of targeted therapies.

The following laboratory parameters obtained at the time of diagnosis: white blood cell (WBC) count, hemoglobin level, hematocrit, platelet count, neutrophil count, red cell distribution width (RDW), C-reactive protein (CRP), erythrocyte sedimentation rate (ESR), serum albumin, total protein, total bilirubin, alkaline phosphatase, aspartate aminotransferase (AST), alanine aminotransferase (ALT), blood urea nitrogen (BUN), creatinine, estimated glomerular filtration rate (eGFR), uric acid, glucose, glycated hemoglobin (HbA1c), calcium, inorganic phosphorus, total cholesterol, triglycerides, high-density lipoprotein (HDL) cholesterol, low-density lipoprotein (LDL) cholesterol, and absolute lymphocyte count. All laboratory tests were routinely conducted at diagnosis according to institutional protocols. Parameters used for calculating nutritional indices were obtained within 1 to 7 days prior to treatment initiation, according to institutional protocol. In all patients, blood samples were collected after an overnight fasting period of at least 8 h, as per routine protocol. Because this was a retrospective study, the timing of laboratory tests followed routine clinical practice and was not additionally standardized for research purposes.

We calculated three pretreatment nutritional indices: CONUT, PNI, and GNRI. In this study, the CONUT score was designated as the primary index of interest, as it integrates nutritional and immune parameters. The PNI and GNRI, both of which have also been validated as meaningful prognostic markers in previous studies [16,17], were included as secondary indices for comparative analysis.

The CONUT score was calculated from serum albumin, total cholesterol, and lymphocyte count, using a validated scoring algorithm (Table S1) [10,11,18].

The PNI was computed [11,19,20,21] as:

PNI = (10 × serum albumin [g/dL]) + (0.005 × total lymphocyte count [/mm^3^])

The GNRI was determined [11,19,22] as:

GNRI = (14.89 × serum albumin [g/dL]) + (41.7 × actual body weight/ideal body weight)

Posttreatment outcomes included mortality at 1 year, 2 years, and overall. Dates of death and most recent follow-up were recorded for each patient. All patients were routinely monitored at the outpatient clinic until death or loss to follow-up. Overall survival was defined as the time interval between the date of diagnosis and either the date of death from any cause or the date of last follow-up.

### 2.4. Study Endpoints

The primary outcome of interest was all-cause mortality within 1 year of diagnosis. The secondary outcome was defined as all-cause mortality occurring within 2 years from the time of diagnosis.

### 2.5. Statistical Analysis

As this was a retrospective study, no a priori sample size estimation was performed. Instead, a post hoc power analysis was conducted using the effect size of the CONUT score obtained from the multivariable Cox regression model for 1-year mortality (HR for CONUT ≥ 3 = 2.071; 95% CI, 1.248–3.437). Based on the corresponding log hazard ratio (β = 0.72785), a two-sided α of 0.05, and a total sample size of 156 patients, the post hoc power was calculated as 89.17%, indicating sufficient statistical power (PASS version 15; NCSS, LLC, Kaysville, UT, USA).

Baseline demographic characteristics, clinical variables, pretreatment laboratory values, and nutritional indices were summarized as mean ± standard deviation (SD) for continuous variables and as counts with percentages for categorical variables. Group comparisons between 1-year survivors and deceased patients were conducted using independent t-tests for continuous data and chi-squared or Fisher’s exact tests for categorical data, as appropriate.

Optimal cut-off values for the CONUT score, PNI, and GNRI were identified using the Contal and O’Quigley method, which identifies the point that maximizes the log-rank test statistic in relation to survival outcomes [23,24]. For the CONUT score, a cut-off of 3 was prespecified based on prior oncology studies that identified ≥ 3 as a marker of malnutrition and poor prognosis [8,9,13,18], and its appropriateness was subsequently verified in our ATC cohort using the Contal and O’Quigley method [23]. This dual strategy ensured comparability with prior literature while also validating the cut-off in our dataset. Kaplan–Meier survival curves were generated, and differences in 1-year and 2-year survival between groups stratified by each index’s optimal cut-off were evaluated using log-rank tests.

To identify predictors of 1-year and 2-year mortality, multivariable Cox proportional hazards models were constructed, incorporating the following variables: high CONUT (≥cut-off), low PNI (≤cut-off), low GNRI (≤cut-off), and albumin (per g/dL). Covariates were selected for inclusion in the multivariable models based on statistical significance in univariable analyses or clinical relevance. To ensure model stability, the number of covariates in the multivariable Cox analysis was constrained according to the rule of one variable per ten outcome events. Based on these criteria, age, tumor size, TNM stage, and surgical treatment were selected for inclusion in the final model.

The predictive performance of each nutritional index and albumin was evaluated by comparing the Harrell’s concordance index (C-index), integrated discrimination improvement (IDI), and net reclassification improvement (NRI) [25]. For each nutritional index, a multivariable model including the index was compared with a baseline model (null model) that included age, tumor size, TNM stage, and surgical treatment. The standard error for the comparison, *p*-value, and 95% confidence interval (CI) were estimated using a bootstrap resampling method with 1000 replicates. It should be noted that these metrics assess model discrimination and reclassification performance and are therefore distinct from measures of association such as ORs or HRs. Specifically, NRI quantifies the improvement in risk classification accuracy when a new model is compared with a baseline model, while IDI evaluated the increase in discriminatory capacity between events and non-events based on predicted probabilities [25].

To explore the prognostic impact of the CONUT score across different treatment modalities, subgroup analyses were performed. Kaplan–Meier survival curves for 1-year overall survival were generated for subgroups with or without surgical treatment, chemotherapy, targeted therapy, and radiation therapy. Exploratory interaction analyses were performed using multivariable Cox proportional hazards models to evaluate potential interactions between pretreatment CONUT score (<3 vs. ≥3) and each treatment modality (surgery, chemotherapy, targeted therapy, and radiation therapy).

Missing data were evaluated across the 20-year cohort. For the main nutritional indices (CONUT, PNI, GNRI), missingness was minimal (<2%). Some laboratory parameters such as TCBI, triglyceride, HDL, LDL, and HbA1c showed higher missing rates, reflecting the retrospective nature of the dataset. Because all variables included in the multivariable models had <5% missing values, a complete-case analysis approach was used. Patients with missing values for covariates of interest were excluded from the corresponding analyses. No imputation was performed.

A two-sided *p*-value < 0.05 was considered statistically significant. Analyses were conducted using SAS (version 9.4; SAS Institute, Cary, NC, USA) and R (version 4.3.2; R Foundation for Statistical Computing, Vienna, Austria).

## 3. Results

### 3.1. Patient Characteristics and Survival Outcomes

**Figure 1:**
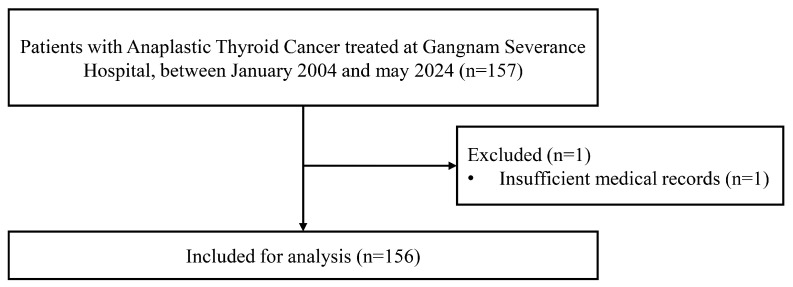
Flowchart of patient inclusion and analysis. Arrows indicate the flow of patient selection and exclusion process.

The final analysis included 156 patients (Figure 1). Table 1 provides the baseline demographic and clinical characteristics of the cohort. The average age was 64.2 years, with males comprising 44.2% of the cohort. Baseline tumor size had a mean value of 5.0 cm. Surgical treatment was performed in 70.5% of patients, chemotherapy in 83.3%, radiation therapy in 82.7%, and targeted therapy in 48.1%. Mortality occurred in 60.3% of patients within 1 year and 69.9% within 2 years, with a median survival period of 7.5 months (IQR, 3.7–16.1 months).

When comparing patients who survived longer than 1 year (non-deceased) with those who died within one year (deceased), several variables differed significantly between the groups (Table 1). Patients in the deceased group were significantly older (66.8 vs. 60.3 years) and had larger tumor sizes (5.3 vs. 4.6 cm) compared to the non-deceased group. A significantly greater proportion of patients in the deceased group had N1 nodal involvement (89.4% vs. 75.8%, *p* = 0.024), distant metastases at diagnosis (M1: 79.8% vs. 46.8%, *p* < 0.001), and advanced disease classified as TNM stage IVc (79.8% vs. 46.8%, *p* < 0.001). Lung metastases were more common in this group (72.3% vs. 40.3%, *p* < 0.001).

Surgical treatment was performed more frequently in the non-deceased group (88.7%) compared to the deceased group (58.5%; *p* < 0.001). Patients in the non-deceased group received a higher cumulative neck radiation dose compared to the deceased group (5044.9 vs. 3785.8 Gy, *p* = 0.014; Table 1). However, there was no significant difference in chemotherapy between the two groups (82.3% vs. 84.0%; *p* = 0.907; Table 1).

### 3.2. Nutritional and Laboratory Parameters Associated with Survival

Pretreatment nutritional indices differed significantly between the deceased and non-deceased groups (Table 2). The deceased group showed higher CONUT scores (2.5 vs. 1.5) and lower PNI (38.0 vs. 41.4) and GNRI (101.8 vs. 107.1) values.

Serum albumin (3.8 vs. 4.1 g/dL; *p* < 0.001) and total protein (6.8 vs. 7.1 g/dL; *p* = 0.025) were reduced in the deceased group. CRP (39.8 vs. 14.9 mg/L) and white blood cell count (12.0 vs. 7.7 × 10^3^/μL) were elevated (*p* < 0.001 for both). Additional differences were noted in inorganic phosphorus, BUN, AST, ALT, and alkaline phosphatase (Table 2).

### 3.3. Cut-Off Point Estimation for Nutritional Markers

Using the Contal and O’Quigley method, the optimal cut-off values were identified as 3 for CONUT, 42 for PNI, and 102 for GNRI. The identified cut-off for the CONUT score aligned with the predefined threshold (3) based on previous studies [8,18]. Although no universally established thresholds exist, our identified cut-offs were consistent with values reported in prior studies [26,27,28,29,30].

### 3.4. Kaplan–Meier Survival Analysis

**Figure 2:**
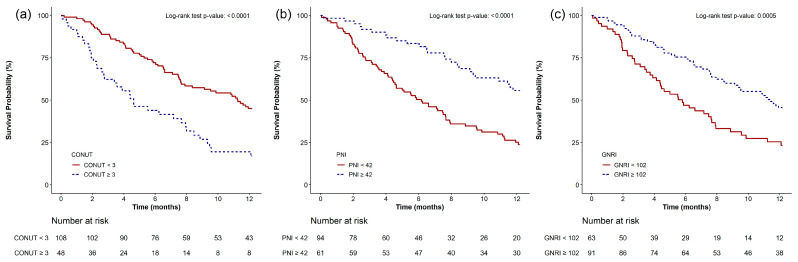
Kaplan–Meier survival curves showing 1-year overall survival according to pretreatment nutritional indices: (**a**) Patients with a Controlling Nutritional Status (CONUT) score < 3 vs. ≥3; (**b**) Patients with a Prognostic Nutritional Index (PNI) > 42 vs. ≤42; (**c**) Patients with a Geriatric Nutritional Risk Index (GNRI) > 102 vs. ≤102. Statistical differences were assessed using log-rank tests.

Kaplan–Meier survival analysis demonstrated significantly increased 1-year mortality among patients with CONUT scores ≥ 3 compared to those with scores < 3 (*p* < 0.0001; Figure 2a). A similar pattern was observed for patients with PNI ≤ 42 or GNRI ≤ 102, both of whom experienced higher 1-year mortality rates (Figure 2b,c). These trends were also observed in the 2-year survival analysis (Figure S1). In exploratory subgroup analyses, a CONUT score ≥ 3 was consistently associated with significantly higher 1-year mortality in patients who underwent surgery (*p* < 0.0001), received chemotherapy (*p* = 0.0006), targeted therapy (*p* = 0.0013), or radiation therapy (*p* = 0.0003) (Figures S2a–S5a).

### 3.5. Independent Prognostic Indicators Associated with One-Year Mortality

Multivariable Cox proportional hazards analysis, adjusted for age, tumor size, TNM stage, and surgical treatment, identified CONUT score ≥ 3 (hazard ratio [HR], 2.071; 95% CI, 1.345–3.187; *p* < 0.001), PNI ≤ 42 (HR, 1.788; 95% CI, 1.092–2.928; *p* = 0.021), and GNRI ≤ 102 (HR, 1.630; 95% CI, 1.075–2.472; *p* = 0.022) as independent predictors of 1-year mortality (Table 3). Results from both univariable and multivariable analyses are provided in Table S2. Variables were tested separately in multivariate models to avoid collinearity. To ensure model stability, the number of covariates in the multivariable Cox analysis was limited based on the rule of one variable per ten outcome events. Among the variables showing significance in univariable analyses, age, tumor size, TNM stage, and surgical treatment were included in the final model due to their clinical importance. Post hoc power analysis based on the observed effect size of the CONUT score (HR = 2.071) demonstrated a statistical power of 89.17%, confirming that the study was adequately powered to detect this association.

In exploratory interaction analyses using multivariable Cox proportional hazards models, a potential interaction was observed between pretreatment CONUT score and surgical treatment for 2-year mortality (*p* = 0.031), and a significant interaction was found between CONUT score and chemotherapy, also for 2-year mortality (*p* = 0.018; Table S3). No significant interaction was observed for 1-year mortality. Given the retrospective design and unmodeled treatment timing or concurrency, these findings should be considered exploratory rather than confirmatory.

### 3.6. Predictive Performance of Nutritional Indices

In the univariable analysis, the PNI showed the highest discriminative ability for predicting 1-year mortality, with a C-index of 0.666, followed by serum albumin (0.665), GNRI (0.629), and CONUT score (0.617; Table 4). Among models based on cut-off values, PNI ≤ 42 had a slightly higher C-index (0.617) than CONUT score ≥ 3 (0.602) and GNRI ≤ 102 (0.596), although pairwise comparisons were not statistically significant.

### 3.7. Added Predictive Value Beyond the Baseline Model

To assess the incremental prognostic value of each nutritional index, we compared each to a baseline model including age, tumor size, TNM stage, and surgery. Incorporating CONUT score ≥ 3 into the baseline model improved its C-index from 0.671 to 0.703 and led to a statistically significant enhancement in discriminatory performance, as reflected by an IDI of 0.035 (95% CI, 0.003–0.087; *p* = 0.032; Table 5). Although PNI ≤ 42, GNRI ≤ 102, and continuous forms of each index did not reach statistical significance, they showed trends toward improved predictive performance, as reflected in increases in C-index, NRI, and IDI values (Table 5).

## 4. Discussion

In this retrospective cohort of ATC patients, those with a CONUT score ≥ 3 had significantly greater 1-year mortality compared to those with a score < 3. After adjustment for major clinical covariates, a CONUT score ≥ 3 remained significantly associated with 1-year mortality. Furthermore, incorporating CONUT ≥ 3 into a baseline prognostic model that included age, tumor size, TNM stage, and surgery significantly improved the model’s predictive performance. These results highlight the prognostic utility of the CONUT score in risk stratification for patients with ATC.

This is, to our knowledge, the first study to demonstrate the prognostic significance of the CONUT score in individuals with ATC. Our findings suggest the independent prognostic value of the pretreatment CONUT score and demonstrate its incremental benefit in risk stratification models. The prognostic relevance of the pretreatment CONUT score in ATC may be explained by two principal mechanisms. First, malnutrition, partially reflected by serum albumin and cholesterol levels, can impair overall physiological reserve and reduce tolerance to aggressive treatments, thereby worsening survival outcomes [31,32,33]. Second, immune dysfunction, indicated by lymphocyte count, may compromise the patient’s ability to respond effectively to cancer therapies, including chemotherapy, targeted therapy, and immunotherapy [1]. As treatment strategies for ATC continue to evolve, baseline immune status may become increasingly important in determining therapeutic response [1,2]. Therefore, both nutritional status and immune competence likely reflect the interplay between host resilience, tumor biology, and treatment responsiveness.

In this study, treatment strategies such as surgery, chemotherapy, targeted therapy, and radiation therapy were carefully considered as potential covariates. Although only surgical treatment was included in the final multivariable model to maintain model stability, exploratory subgroup and interaction analyses confirmed that the prognostic association of the CONUT score remained consistent across treatment modalities. These findings suggest that baseline nutritional and immune status influence treatment tolerance and response, thereby reinforcing the biological plausibility of the observed prognostic relationship. Thus, the CONUT score complements, rather than replaces, treatment-related factors by capturing host vulnerability not reflected in conventional prognostic variables.

We selected the CONUT score as the primary nutritional index in this study because it comprehensively reflects both immune competence and overall nutritional status. Unlike PNI and GNRI, it incorporates serum total cholesterol, which has increasingly been recognized as a surrogate indicator of systemic inflammation and metabolic reserve [31,32,33]. Hypocholesterolemia in cancer patients has been reported as a marker of aggressive tumor biology, including increased cholesterol consumption by rapidly dividing cells, cytokine-driven suppression of hepatic synthesis, and altered systemic lipid metabolism [34,35,36]. This phenomenon likely reflects a broader catabolic and inflammatory state, especially relevant in malignancies such as ATC, where systemic deterioration is common. Cholesterol functions as a structural lipid and plays critical roles in steroidogenesis, membrane raft formation, and T cell receptor signaling [35,37]. Accordingly, decreased cholesterol levels may signify impaired immunometabolic capacity to withstand tumor progression and treatment-related stress. While Yu et al. previously reported the prognostic relevance of PNI in ATC, their analysis was limited to that single index and involved a relatively small sample size [1]. In contrast, the present study included a larger cohort and directly compared the predictive value of the CONUT score, PNI, and GNRI using multiple statistical metrics. Of the three indices, the CONUT score consistently yielded the greatest enhancement in model performance when added to the baseline model. This finding was supported by a statistically significant improvement in the IDI (*p* = 0.032), indicating enhanced overall model discrimination [25]. Although the increase in Harrell’s c-index, from 0.671 to 0.703, did not reach statistical significance (*p* = 0.100), it suggests a trend toward better risk discrimination. The NRI, which measures reclassification accuracy [25], also showed a trend toward improvement. In contrast, while the PNI and GNRI demonstrated similar trends in IDI and NRI, their improvements were not significant. Overall, a CONUT score ≥ 3 yielded the most consistent enhancement across discrimination metrics, supporting its clinical utility as an additive prognostic marker in patients with ATC.

Beyond statistical performance, the clinical relevance of our findings deserves emphasis. The CONUT score can be readily calculated from routine laboratory data, making it practical for early risk stratification in patients with ATC, where timely treatment decisions are critical and advanced molecular profiling may not always be available. Compared with PNI, which reflects protein reserve and lymphocyte-mediated immunity, and GNRI, which is strongly influenced by body weight, CONUT offers a more comprehensive assessment by additionally incorporating serum cholesterol [31]. This metabolic component may capture systemic catabolism and tumor-driven lipid consumption, processes that are particularly relevant in rapidly progressive cancers such as ATC [32,33,38]. These differences likely explain the modest discrepancies in predictive performance observed among the indices in our cohort. Although our analysis should be regarded as exploratory due to its retrospective single-center design, the consistent association between CONUT and survival highlights its potential as a clinically meaningful and easily implementable prognostic tool. Future multicenter, prospective studies are warranted to validate whether nutritional or immunologic interventions guided by CONUT stratification could improve outcomes in this challenging malignancy.

This study identified a pretreatment CONUT score of ≥3 as an independent predictor of increased 1-year mortality in patients with ATC, with robust associations even after adjustment for clinical covariates. By integrating nutritional, immune, and metabolic information, the CONUT score provides a more integrative assessment than other nutritional markers. Unlike prior studies that focused on a single index or lacked validation against established clinical parameters [1,9], the present study systematically compared three indices in a relatively large, well-characterized cohort using robust performance metrics, including the C-index, IDI, and NRI. This comparative framework enhances the translational relevance of our findings and supports the incorporation of immuno-nutritional assessment into routine prognostic evaluation. However, given the limited sample size, retrospective single-center design, and temporal heterogeneity in treatment over the 20-year inclusion period, our findings should be interpreted with caution. These results should be considered preliminary, and confirmation in larger, multicenter cohorts treated with contemporary therapeutic strategies is warranted.

This study does have several limitations. First, due to its retrospective observational design, the analysis may be affected by residual confounding, despite adjustments for relevant clinical variables in the multivariable analysis. Second, it was a single-center study, and the generalizability of the results can be restricted. Third, although we identified optimal cut-off values for each nutritional index using a validated statistical method, these thresholds are not universally established, and different cut-off values may yield different results. Fourth, we did not assess temporal changes in nutritional indices during treatment, which may provide additional prognostic or predictive insights [39]. Although albumin, cholesterol, and lymphocyte counts are easily and routinely measured in clinical practice, the present study focused on the prognostic utility of baseline values obtained prior to treatment initiation. Evaluating dynamic changes would require a longitudinal design with serial assessments, which was beyond the scope of this study but warrants investigation in future prospective research. Fifth, the present study did not assess whether nutritional or immunologic interventions could influence clinical outcomes, highlighting the need for future prospective interventional research to explore this possibility. Sixth, we classified patients based on one-year survival to evaluate the prognostic performance of nutritional indices in long-term outcomes. However, due to the extremely short median survival in ATC, this led to a significant imbalance in group sizes, which may have affected statistical power and generalizability. Seventh, detailed treatment duration for each chemotherapy regimen could not be analyzed due to the retrospective design and heterogeneity of treatment practices across the 20-year study period. This may have influenced outcome interpretation and should be considered when evaluating our findings. Lastly, this study lacked mutational data, despite its growing importance in guiding targeted therapy for ATC [40]. Given the 20-year inclusion period, treatment regimens were heterogeneous and evolved considerably over time. These temporal changes and the relatively small size should be considered when interpreting the results. Nevertheless, by demonstrating the prognostic relevance of the CONUT score in ATC, our study provides a preliminary foundation for future multicenter research to validate its utility within the context of modern therapeutic strategies.

## 5. Conclusions

This study identified a pretreatment CONUT score of ≥3 as an independent predictor of increased 1-year mortality in patients with ATC. This association remained robust even after adjusting for key clinical variables. Furthermore, incorporating the CONUT score into baseline prediction models significantly improved their ability to predict 1-year mortality. While these findings are exploratory, they remain clinically meaningful, as they highlight the potential utility of the CONUT score as a simple and readily available prognostic tool. Confirmation in larger, multicenter cohorts with contemporary treatment approaches is warranted to establish its role in current ATC management.

## Figures and Tables

**Table 1:** Baseline demographic and clinical characteristics of the study population according to the survival status at 1 year after initial diagnosis.

Characteristics	Overall (n = 156)	1-Year Survival Status		*p* Value
		Non-Deceased (n = 62)	Deceased (n = 94)	
Age (yr)	64.2 (11.3)	60.3 (11.8)	66.8 (10.2)	<0.001
Male sex	69 (44.2%)	31 (50.0%)	38 (40.4%)	0.239
BMI (kg/m^2^)	23.6 (3.2)	24.1 (3.6)	23.4 (2.9)	0.188
Tumor size (cm)	5.0 (2.3)	4.6 (2.2)	5.3 (2.4)	0.049
T stage				0.249
T2	11 (7.1%)	6 (9.7%)	5 (5.3%)	
T3a	5 (3.2%)	3 (4.8%)	2 (2.15)	
T3b	17 (10.9%)	9 (14.5%)	8 (8.5%)	
T4	123 (78.9%)	44 (71.0%)	79 (84.0%)	
N stage				
N1	131 (84.0%)	47 (75.8%)	84 (89.4%)	0.024
M stage				
M1	104 (66.7%)	29 (46.8%)	75 (79.8%)	<0.001
TNM Staging				<0.001
TNM stage IVa	11 (7.1%)	9 (14.5%)	2 (2.1%)	
TNM stage IVb	41 (26.3%)	24 (38.7%)	17 (18.1%)	
TNM stage IVc	104 (66.7%)	29 (46.8%)	75 (79.8%)	
Metastasis				
Lung	93 (59.6%)	25 (40.3%)	68 (72.3%)	<0.001
Bone	31 (19.9%)	8 (12.9%)	23 (24.5%)	0.077
Brain	17 (10.9%)	5 (8.1%)	12 (12.8%)	0.356
Pancreas	3 (1.9%)	0 (0)	3 (3.2%)	0.277
Adrenal gland	3 (1.9%)	0 (0)	3 (3.2%)	0.277
Liver	6 (3.9%)	2 (3.2%)	4 (4.3%)	>0.999
Mediastinum	10 (6.4%)	3 (4.8%)	7 (7.5%)	0.741
Surgery	110 (70.5%)	55 (88.7%)	55 (58.5%)	<0.001
Type of Surgery				0.111
Excisional biopsy	22 (19.3%)	7 (12.7%)	15 (25.4%)	
Debulking	40 (35.1%)	18 (32.7%)	22 (37.3%)	
Complete resection	52 (45.6%)	30 (54.6%)	22 (37.3%)	
Chemotherapy	130 (83.3%)	51 (82.3%)	79 (84.0%)	0.907
First chemotherapy regimen				
Adriamycin	15 (11.5%)	5 (9.8%)	10 (12.7%)	
Cisplatin	4 (3.1%)	2 (3.9%)	2 (2.5%)	
Epirubicin	1 (0.8%)	0 (0)	1 (1.3%)	
Paclitaxel	111 (85.4%)	44 (86.3%)	67 (84.8%)	
Second chemotherapy regimen				
Adriamycin	3 (2.3%)	1 (2.0%)	2 (2.5%)	
Carboplatin	2 (1.5%)	1 (2.0%)	1 (1.3%)	
Paclitaxel	9 (6.9%)	4 (7.8%)	5 (6.3%)	
Targeted therapy	75 (48.1%)	28 (45.2%)	47 (50.0%)	0.554
First-line targeted therapy regimen, Lenvima	61 (81.3%)	24 (85.7%)	37 (78.7%)	
First-line targeted therapy regimen, Nexavar	14 (18.7%)	4 (14.3%)	10 (21.3%)	
Second-line targeted therapy regimen, Lenvima	3 (4.0%)	1 (3.6%)	2 (4.3%)	
Radiation therapy	129 (82.7%)	54 (87.1%)	75 (79.8%)	0.238
Neck radiation dose (Gy)	4287.8 (2955.5)	5044.9 (3302.3)	3785.8 (2600.3)	0.014
Radiation therapy, bone	4 (3.1%)	1 (1.9%)	3 (4.0%)	
Radiation therapy, brain	6 (4.7%)	2 (3.7%)	4 (5.3%)	
Radiation therapy, lung	4 (3.1%)	2 (3.7%)	2 (2.7%)	
Radiation therapy, iliac	1 (0.8%)	1 (1.9%)	0 (0)	
Radiation therapy, spine	6 (4.7%)	1 (1.9%)	5 (6.7%)	
Other site radiation dose (Gy)	4434.2 (1842.5)	4292.9 (1772.6)	4516.7 (1954.8)	0.807

Patients were classified into two groups based on 1-year survival: those who survived beyond one year (non-deceased) and those who died within one year (deceased). Values are presented as mean (standard deviation) or number (%). Percentages are calculated based on the number of patients in each column group (overall, non-deceased, and deceased). Abbreviations: BMI, body mass index (calculated as weight in kilograms divided by height in meters squared); Gy: Gray; TNM, Tumor-Node-Metastasis staging.

**Table 2:** Pretreatment Laboratory Data and Nutritional Indices.

Characteristics	Overall (n = 156)	1-Year Survival Status		*p* Value
		Non-Deceased (n = 62)	Deceased (n = 94)	
Controlling Nutritional Status (CONUT) score	2.1 (2.0)	1.5 (1.5)	2.5 (2.3)	0.001
CONUT < 3	108 (69.2)	51 (82.3)	57 (60.6)	0.004
CONUT ≥ 3	48 (30.8)	11 (17.7)	37 (39.4)	
Prognostic nutritional index (PNI)	39.3 (5.4)	41.4 (4.4)	38.0 (5.6)	<0.001
PNI > 42	61 (39.4)	36 (59.0)	25 (26.6)	<0.001
PNI ≤ 42	94 (60.7)	25 (41.0)	69 (73.4)	
Geriatric Nutritional Risk Index (GNRI)	103.9 (10.8)	107.1 (9.8)	101.8 (11.0)	0.003
GNRI > 102	91 (59.1)	44 (72.1)	47 (50.5)	0.008
GNRI ≤ 102	63 (40.9)	17 (27.9)	46 (49.5)	
Albumin (g/dL)	3.9 (0.5)	4.1 (0.4)	3.8 (0.6)	<0.001
Total cholesterol (mg/dL)	170.3 (42.6)	175.4 (43.5)	166.9 (41.9)	0.221
Lymphocyte (10^3^/μL)	1.7 (0.6)	1.8 (0.5)	1.7 (0.7)	0.454
Calcium (mg/dL)	8.8 (0.8)	8.8 (0.7)	8.8 (0.8)	0.985
Inorganic Phosphorus (mg/dL)	3.7 (0.7)	3.9 (0.7)	3.6 (0.6)	0.022
Glucose (mg/dL)	124.2 (35.5)	122.3 (30.8)	125.5 (38.4)	0.585
BUN (mg/dL)	15.5 (5.8)	14.2 (5.0)	16.3 (6.2)	0.028
Creatinine (mg/dL)	0.7 (0.4)	0.7 (0.2)	0.8 (0.5)	0.810
Uric acid (mg/dL)	4.5 (1.5)	4.6 (1.5)	4.4 (1.5)	0.346
Total protein (g/dL)	6.9 (0.7)	7.1 (0.6)	6.8 (0.7)	0.025
Total bilirubin (mg/dL)	0.6 (0.2)	0.6 (0.2)	0.6 (0.2)	0.266
Alkaline phosphatase (IU/L)	92.8 (44.5)	81.2 (22.9)	100.4 (53.1)	0.002
Aspartate aminotransferase (IU/L)	22.0 (8.3)	24.1 (9.3)	20.6 (7.4)	0.015
Alanine aminotransferase (IU/L)	18.9 (12.3)	22.3 (15.8)	16.6 (8.6)	0.011
Triglyceride (mg/dL)	126.8 (76.9)	119.7 (75.7)	135.8 (78.5)	0.329
HDL-cholesterol (mg/dL)	45.1 (12.7)	46.6 (10.6)	42.8 (15.2)	0.218
LDL-cholesterol (mg/dL)	109.2 (31.1)	108.8 (32.1)	109.9 (30.0)	0.880
HbA1c (%)	6.6 (1.1)	6.4 (1.0)	6.7 (1.1)	0.469
White blood cell (10^3^/μL)	10.3 (8.0)	7.7 (2.6)	12.0 (9.7)	<0.001
Hemoglobin (g/dL)	12.7 (1.7)	13.2 (1.5)	12.4 (1.8)	0.009
Hematocrit (%)	38.3 (4.9)	39.6 (4.2)	37.4 (5.1)	0.007
Red cell distribution width (%)	13.0 (1.2)	12.8 (1.1)	13.2 (1.2)	0.049
Platelet (10^3^/μL)	288.5 (117.7)	277.9 (96.9)	295.4 (129.6)	0.338
Neutrophil (10^3^/μL)	7.5 (7.4)	5.1 (2.4)	9.1 (8.9)	<0.001
Erythrocyte Sedimentation Rate (mm/hr)	44.6 (29.4)	42.0 (28.5)	46.3 (30.2)	0.472
C-Reactive Protein (mg/L)	30.1 (44.1)	14.9 (28.1)	39.8 (49.6)	<0.001
eGFR (mL/min/1.73 m^2^)	101.9 (30.6)	100.2 (25.1)	103.0 (33.9)	0.545

Patients were classified into two groups based on 1-year survival: those who survived beyond one year (non-deceased) and those who died within one year (deceased). Values are presented as mean (standard deviation) or number (%). Percentages are calculated based on the number of patients in each column group (overall, non-deceased, and deceased). Abbreviations: CONUT, Controlling Nutritional Status; PNI, Prognostic Nutritional Index; GNRI, Geriatric Nutritional Risk Index; eGFR, estimated glomerular filtration rate; HbA1c, glycated hemoglobin.

**Table 3:** The multivariable Cox proportional hazard model of factors predicting the 1-year and 2-year mortality.

Variable	1-Year Mortality		2-Year Mortality	
	HR (95% CI)	*p* Value	HR (95% CI)	*p* Value
CONUT				
<3	ref		ref	
≥3	2.071 (1.345–3.187)	<0.001	2.040 (1.356–3.068)	0.001
PNI				
>42	ref		ref	
≤42	1.788 (1.092–2.928)	0.021	1.779 (1.135–2.788)	0.0121
GNRI				
>102	ref		ref	
≤102	1.630 (1.075–2.472)	0.022	1.528 (1.034–2.259)	0.034
Albumin (per g/dL)	0.436 (0.288–0.660)	<0.001	0.477 (0.323–0.702)	<0.001

Values are hazard ratios (HRs) with 95% confidence intervals (CIs). Individual multivariable Cox models were performed for each variable, adjusting for age, tumor size, TNM staging, and surgery. (Age, tumor size, TNM staging, and surgery were used as covariates.). Abbreviations: CONUT, Controlling Nutritional Status; PNI, Prognostic Nutritional Index; GNRI, Geriatric Nutritional Risk Index; ref, reference category used for comparison in Cox proportional hazards regression analysis.

**Table 4:** Univariable prognostic utility of the Controlling Nutritional Status score, Prognostic Nutritional Index, Geriatric Nutritional Risk Index, and serum albumin concentration in predicting 1-year mortality of patients with anaplastic thyroid cancer.

Variable	Harrell’s C-Index (95% CI)	*p* Value		
**Classification by the optimal cut-off values**		**vs. CONUT ≥ 3**	**vs. PNI ≤ 42**	**vs. GNRI ≤ 102**
CONUT ≥ 3	0.602 (0.554–0.65)	Ref	0.6714	0.8756
PNI ≤ 42	0.617 (0.568–0.666)	0.6714	Ref	0.5563
GNRI ≤ 102	0.596 (0.544–0.647)	0.8756	0.5563	Ref
**Continuous variable**		**vs. CONUT**	**vs. PNI**	**vs. GNRI**
CONUT	0.617 (0.558–0.675)	Ref	0.251	0.795
PNI	0.666 (0.605–0.726)	0.251	Ref	0.410
GNRI	0.629 (0.565–0.693)	0.795	0.410	Ref
Albumin (g/dL)	0.665 (0.606–0.724)	0.090	0.991	0.423

Values are Harrell’s C-index (95% confidence interval). *p*-values indicate pairwise comparison between models. Abbreviations: CONUT, Controlling Nutritional Status; PNI, Prognostic Nutritional Index; GNRI, Geriatric Nutritional Risk Index.

**Table 5:** Comparison of Predictive Discrimination Metrics for Nutritional Indices Versus Baseline Model for One-Year Mortality in Patients with Anaplastic Thyroid Cancer (Multivariable Analysis).

	CONUT (Cut-off)			CONUT (Continuous)		
	**Null model**	**Null model + CONUT ≥ 3**	***p* value**	**Null model**	**Null model + CONUT (continuous)**	***p* value**
	**Predictive ability (95% CI)**	**Predictive ability (95% CI)**		**Predictive ability (95% CI)**	**Predictive ability (95% CI)**	
Harrell’s c index	0.671 (0.612–0.729)	0.703 (0.648–0.757)	0.100	0.671 (0.612–0.729)	0.698 (0.645–0.752)	0.146
NRI	-	0.160 (−0.045–0.321)	0.100	-	0.165 (−0.105–0.323)	0.194
IDI	-	0.035 (0.003–0.087)	0.032	-	0.027 (−0.003–0.068)	0.074
	**PNI (cut-off)**			**PNI (continuous)**		
	**Null model**	**Null model + PNI≤ 42**	***p* value**	**Null model**	**Null model + PNI (continuous)**	***p* value**
	**Predictive ability (95% CI)**	**Predictive ability (95% CI)**		**Predictive ability (95% CI)**	**Predictive ability (95% CI)**	
Harrell’s c index	0.671 (0.612–0.729)	0.691 (0.634–0.748)	0.633	0.671 (0.612–0.729)	0.707 (0.651–0.762)	0.402
NRI	-	0.291 (−0.024–0.459)	0.074	-	0.138 (−0.059–0.336)	0.126
IDI	-	0.025 (−0.002–0.083)	0.090	-	0.036 (−0.002–0.101)	0.076
	**GNRI (cut-off)**			**GNRI (continuous)**		
	**Null model**	**Null model + GNRI ≤ 102**	***p* value**	**Null model**	**Null model + GNRI (continuous)**	***p* value**
	**Predictive ability (95% CI)**	**Predictive ability (95% CI)**		**Predictive ability (95% CI)**	**Predictive ability (95% CI)**	
Harrell’s c index	0.671 (0.612–0.729)	0.695 (0.64–0.75)	0.547	0.671 (0.612–0.729)	0.711 (0.656–0.766)	0.312
NRI	-	0.244 (−0.109–0.396)	0.132	-	0.123 (−0.109–0.321)	0.234
IDI	-	0.020 (−0.004–0.071)	0.136	-	0.034 (−0.003–0.096)	0.082

Abbreviations: CONUT, Controlling Nutritional Status; PNI, Prognostic Nutritional Index; GNRI, Geriatric Nutritional Risk Index; NRI, net reclassification improvement; IDI, integrated discrimination improvement. Null models include age, tumor size, TNM stage, and surgery. C-index values are presented with 95% confidence intervals. Net Reclassification Improvement (NRI) quantifies the improvement in risk classification accuracy when a new model is compared to a baseline model. Integrated Discrimination Improvement (IDI) measures the improvement in a model’s ability to distinguish between events and non-events by comparing the difference in predicted probabilities between two models. Note: Table 5 presents comparative predictive performance metrics and does not include odds ratios (ORs) or hazards ratios (HRs). Harrell’s c-index, NRI, and IDI assess discrimination and reclassification performance of the models, not associations between variables. *p*-values are for comparison with the null model. All statistical tests were two-tailed, and *p* < 0.05 was considered statistically significant and 0.05 ≤ *p* < 0.2 was considered a trend toward significance to increase the sensitivity to detect potential selection bias.

## Data Availability

The data are available from the corresponding author upon reasonable request.

## Supplementary Materials

The following supporting information can be downloaded at: https://www.mdpi.com/article/10.3390/cancers17203344/s1, Methods S1: Treatment protocol for anaplastic thyroid cancer; Figure S1: Kaplan–Meier survival curves depicting 2-year survival according to pretreatment nutritional indices: (a) Patients with a Controlling Nutritional Status (CONUT) score < 3 vs. ≥3, (b) Patients with a Prognostic Nutritional Index (PNI) > 42 vs. ≤42, (c) Patients with a Geriatric Nutritional Risk Index (GNRI) > 102 vs. ≤102. Group comparisons were performed using the log-rank test; Figure S2: Kaplan–Meier survival curves for 1-year overall survival according to pretreatment Controlling Nutritional Status (CONUT) score in subgroup analyses: (a) Patients who underwent surgery; (b) Patients who did not undergo surgery; Figure S3: Kaplan–Meier survival curves for 1-year overall survival according to pretreatment Controlling Nutritional Status (CONUT) score in subgroup analyses: (a) Patients who received chemotherapy; (b) Patients who did not receive chemotherapy; Figure S4: Kaplan–Meier survival curves for 1-year overall survival according to pretreatment Controlling Nutritional Status (CONUT) score in subgroup analyses: (a) Patients who received targeted therapy; (b) Patients who did not receive targeted therapy; Figure S5: Kaplan–Meier survival curves for 1-year overall survival according to pretreatment Controlling Nutritional Status (CONUT) score in subgroup analyses: (a) Patients who received radiation therapy; (b) Patients who did not receive radiation therapy; Table S1: The Controlling Nutritional Status (CONUT) scoring system; Table S2: Univariable and multivariable Cox proportional hazards regression analyses for 1-year mortality in patients with anaplastic thyroid cancer; Table S3: Interaction analysis of pretreatment Controlling Nutritional Status (CONUT) score and each treatment modality (surgical treatment, chemotherapy, targeted therapy, radiation therapy) for predicting 1-year and 2-year mortality in anaplastic thyroid cancer.

## Abbreviations

The following abbreviations are used in this manuscript:

ATC	Anaplastic thyroid cancer
CONUT	Controlling Nutritional Status
PNI	Prognostic Nutritional Index
GNRI	Geriatric Nutritional Risk Index
BMI	Body mass index
TNM	Tumor-Node-Metastasis
WBC	White blood cell
RDW	Red cell distribution width
CRP	C-reactive protein
ESR	Erythrocyte sedimentation rate
AST	Aspartate aminotransferase
ALT	Alanine aminotransferase
BUN	Blood urea nitrogen
eGFR	Estimated glomerular filtration rate
HbA1c	Glycated hemoglobin
HDL	High-density lipoprotein
LDL	Low-density lipoprotein
C-index	Concordance index
IDI	Integrated discrimination improvement
NRI	Net reclassification improvement
CI	Confidence interval
ROC	Receiver operating characteristic
