# Supplementary material for: Prognostic Impact of the Pretreatment Controlling Nutritional Status (CONUT) Score in Anaplastic Thyroid Cancer: A Retrospective Cohort Study
Source: Cancers (Basel). 2025 Oct 16;17(20):3344. doi: 10.3390/cancers17203344 (PMC12564293; doi:10.3390/cancers17203344)
Supplement: Supplementary file 1 [file cancers-17-03344-s001.zip › supplementary data_cancers_ver 1.0.pdf]

List of Supplementary Materials

Title	Page
**Supplemental Methods S1.** Treatment protocol for Anaplastic Thyroid Cancer	2
**Supplemental Table S1.** The Controlling Nutritional Status (CONUT) scoring system	3
**Supplementary Figure S1.** Kaplan–Meier curves for 2-year survival stratified by pretreatment nutritional indices. (a) Patients with a Controlling Nutritional Status (CONUT) score <3 vs. ≥3, (b) Patients with a Prognostic Nutritional Index (PNI) >42 vs. ≤42, (c) Patients with a Geriatric Nutritional Risk Index (GNRI) >102 vs. ≤102. Statistical comparisons between groups were performed using the log-rank test.	4
**Supplementary Figure S2.** Kaplan–Meier survival curves for 1-year overall survival according to pretreatment Controlling Nutritional Status (CONUT) score in subgroup analyses: (a) Patients who underwent surgery; (b) Patients who did not undergo surgery.	5
**Supplementary Figure S3.** Kaplan–Meier survival curves for 1-year overall survival according to pretreatment Controlling Nutritional Status (CONUT) score in subgroup analyses: (a) Patients who received chemotherapy; (b) Patients who did not receive chemotherapy.	6
**Supplementary Figure S4.** Kaplan–Meier survival curves for 1-year overall survival according to pretreatment Controlling Nutritional Status (CONUT) score in subgroup analyses: (a) Patients who received targeted therapy; (b) Patients who did not receive targeted therapy.	7
**Supplementary Figure S5.** Kaplan–Meier survival curves for 1-year overall survival according to pretreatment Controlling Nutritional Status (CONUT) score in subgroup analyses: (a) Patients who received radiation therapy; (b) Patients who did not receive radiation therapy.	8
**Supplemental Table S2.** Cox proportional hazard model of 1-year mortality.	9
**Supplemental Table S3.** Interaction analysis of pretreatment Controlling Nutritional Status (CONUT) score and each treatment modality (surgical treatment, chemotherapy, targeted therapy, radiation therapy) for predicting 1-year and 2-year mortality in anaplastic thyroid cancer.	12

Supplemental Methods S1. Treatment protocol for Anaplastic Thyroid Cancer

Initial Evaluation and Induction Therapy

Patients with resectable primary tumors and no distant metastases were considered surgical candidates. All patients initially received one cycle of paclitaxel (70 mg/m^2 weekly for 3 weeks, followed by a 1-week break) in combination with intensity-modulated radiotherapy (IMRT, 3960 cGy). After 6 weeks, neck imaging (PET-CT or MRI) was performed to reassess resectability.

Surgical Candidates

If surgery was deemed feasible, patients received an additional preoperative cycle of paclitaxel (70 mg/m^2) with IMRT (2640 cGy), followed by surgical resection. Postoperatively, up to three additional cycles of paclitaxel (without IMRT) were administered. Routine imaging and laboratory assessments were conducted every two cycles to evaluate treatment response.

Unresectable Disease

Patients with unresectable tumors after initial therapy continued paclitaxel with IMRT. Imaging assessments were repeated every two cycles. Upon disease progression, lenvatinib was initiated at 10 mg/day and escalated to 20 mg/day and 24 mg/day if progression persisted. The initial low dose was chosen due to prior clinical experience with significant toxicities (e.g., leukopenia, muscle weakness) at higher starting doses.

Targeted Therapy

For progressive disease, lenvatinib was the preferred tyrosine kinase inhibitor (TKI). Sorafenib was used prior to the establishment of this protocol.

Limitations on Paclitaxel Use

Postoperative paclitaxel was limited to a maximum of six cycles per patient.

Supplemental Table S1. The Controlling Nutritional Status (CONUT) scoring system

**Parameters**	**Degree of malnutrition**			
	**Normal**	**Mild**	**Moderate**	**Severe**
**Serum albumin (g/dL)**	≥ 3.5	3.0–3.4	2.5–2.9	< 2.5
Score	0	2	4	6
**Total lymphocyte count (/mm^3)**	≥ 1600	1200–1599	800–1199	< 800
Score	0	1	2	3
**Total cholesterol (mg/dL)**	≥ 180	140–179	100–139	< 100
Score	0	1	2	3
**CONUT score (total)**	0–1	2–4	5–8	9–12

CONUT, Controlling Nutritional Status.

Supplementary Figure S1. Kaplan–Meier curves for 2-year survival stratified by pretreatment nutritional indices. (a) Patients with a Controlling Nutritional Status (CONUT) score <3 vs. ≥3, (b) Patients with a Prognostic Nutritional Index (PNI) >42 vs. ≤42, (c) Patients with a Geriatric Nutritional Risk Index (GNRI) >102 vs. ≤102. Statistical comparisons between groups were performed using the log-rank test.

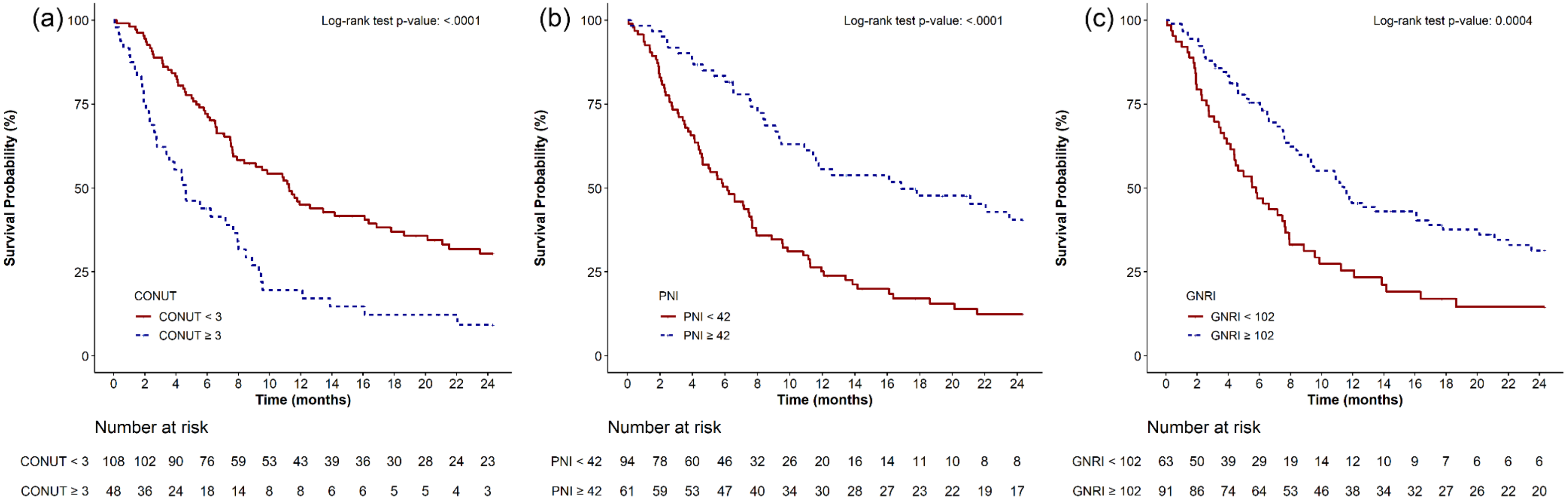

Supplementary Figure S2. Kaplan–Meier survival curves for 1-year overall survival according to pretreatment Controlling Nutritional Status (CONUT) score in subgroup analyses: (a) Patients who underwent surgery; (b) Patients who did not undergo surgery.

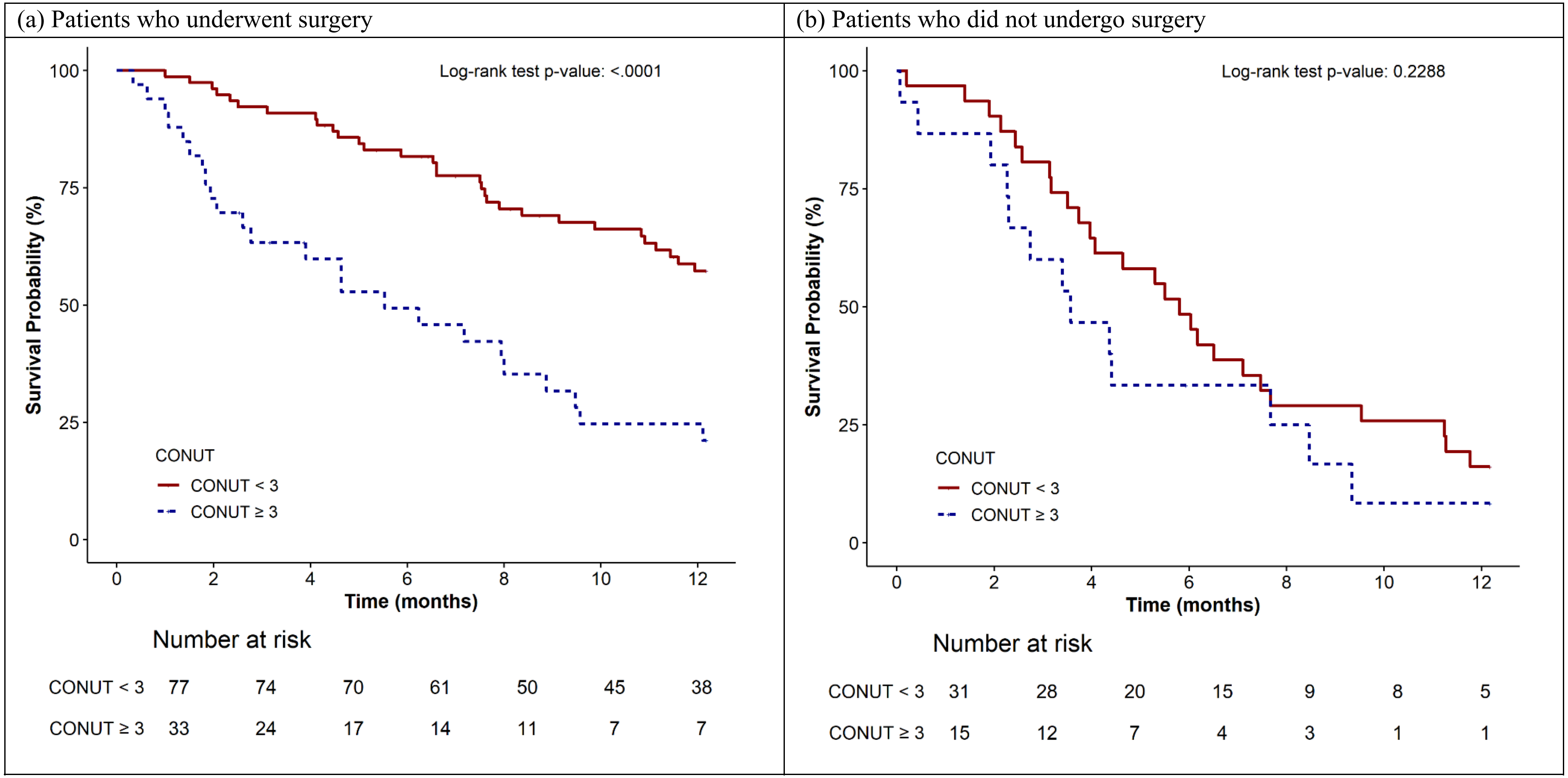

Supplementary Figure S3. Kaplan–Meier survival curves for 1-year overall survival according to pretreatment Controlling Nutritional Status (CONUT) score in subgroup analyses: (a) Patients who received chemotherapy; (b) Patients who did not receive chemotherapy.

(a) Patients who received chemotherapy | (b) Patients who did not receive chemotherapy

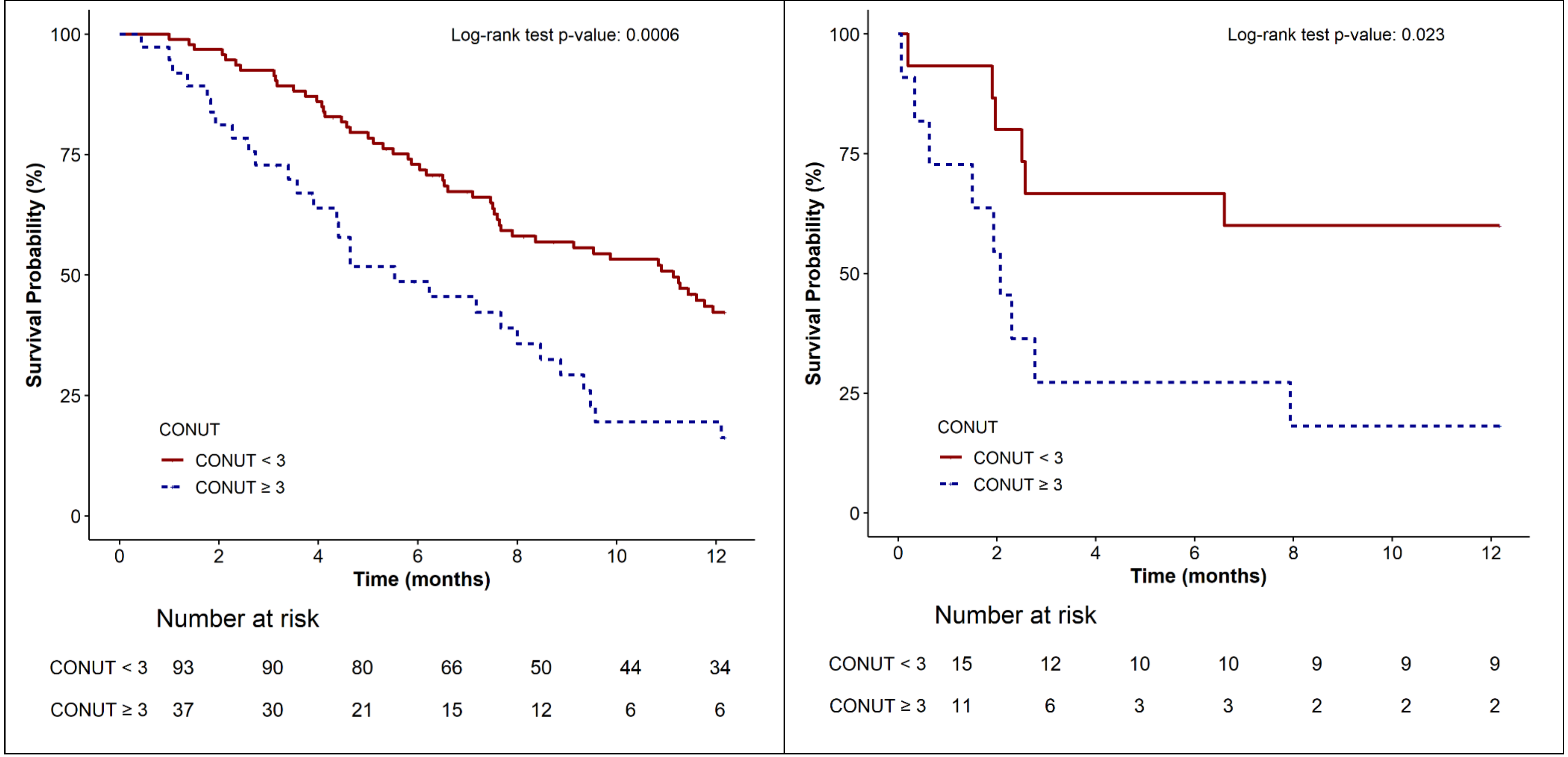

Supplementary Figure S4. Kaplan–Meier survival curves for 1-year overall survival according to pretreatment Controlling Nutritional Status (CONUT) score in subgroup analyses: (a) Patients who received targeted therapy; (b) Patients who did not receive targeted therapy.

(a) Patients who received targeted therapy | (b) Patients who did not receive targeted therapy

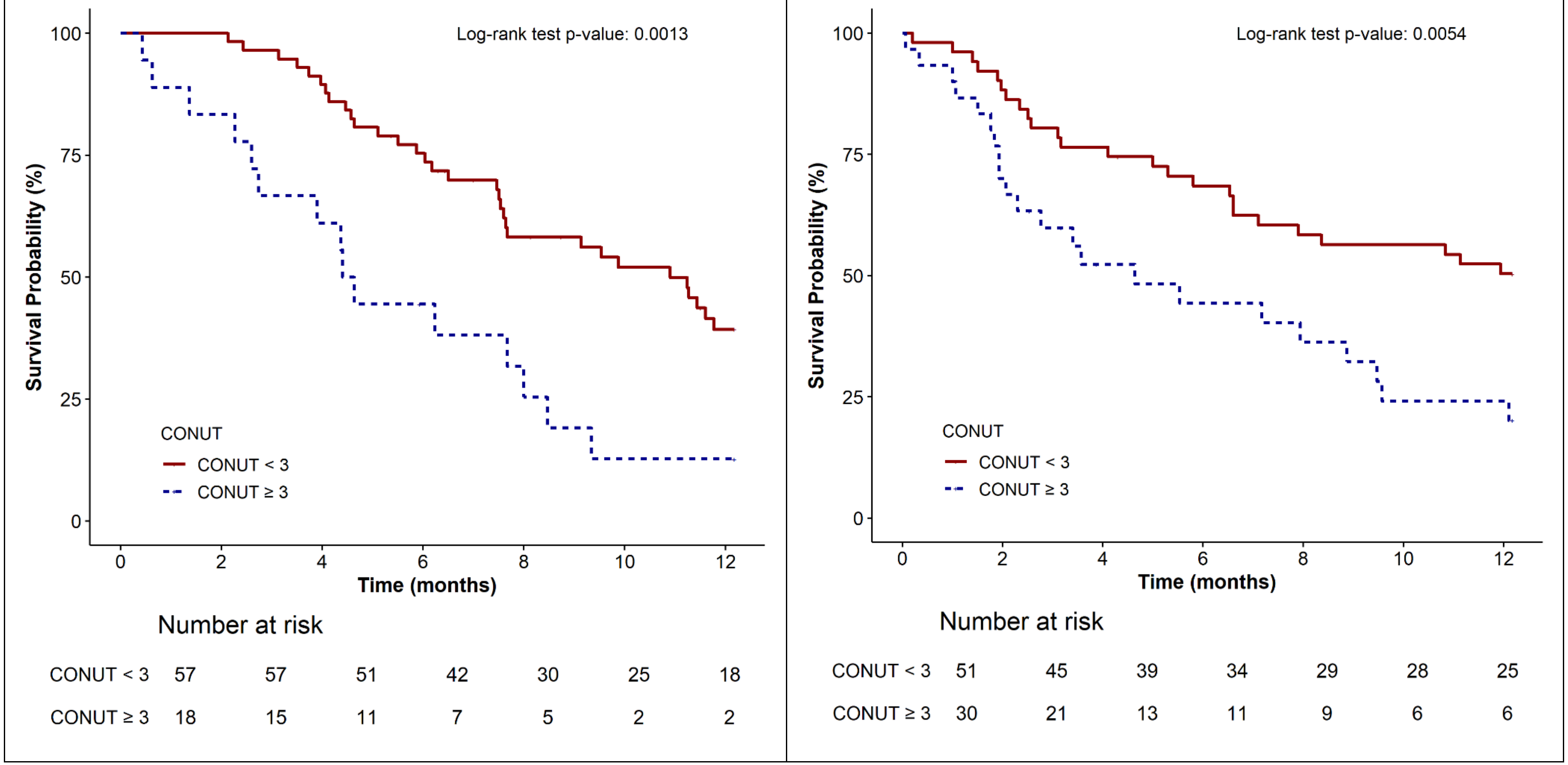

Supplementary Figure S5. Kaplan–Meier survival curves for 1-year overall survival according to pretreatment Controlling Nutritional Status (CONUT) score in subgroup analyses: (a) Patients who received radiation therapy; (b) Patients who did not receive radiation therapy.

(a) Patients who received radiation therapy

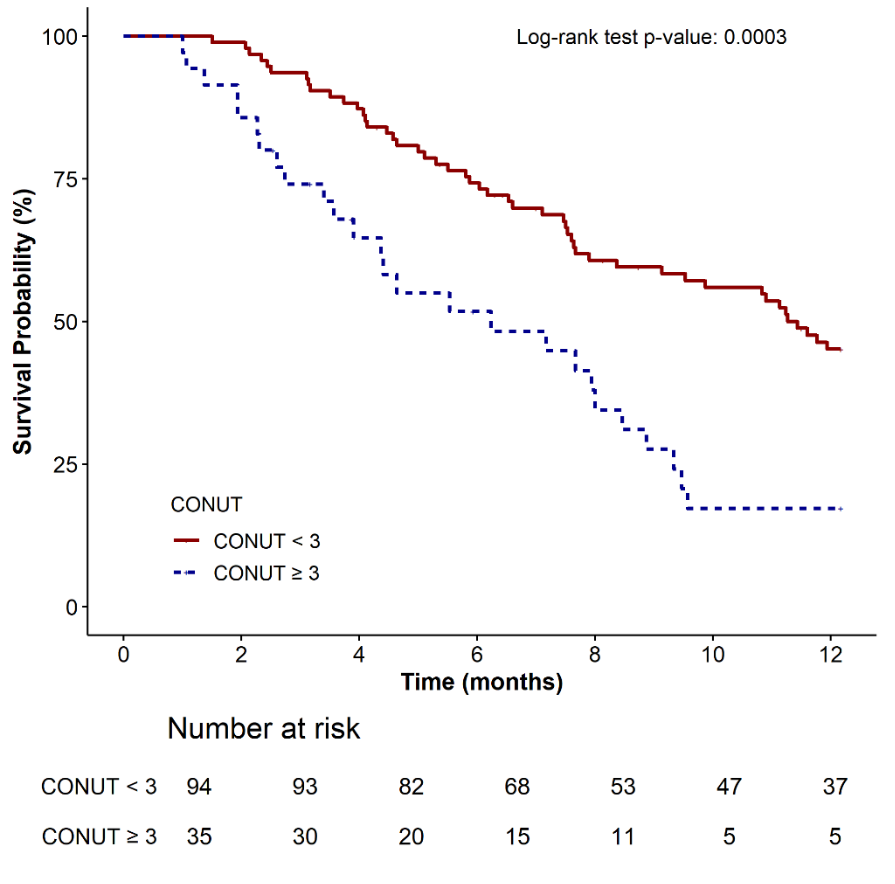

(b) Patients who did not receive radiation therapy

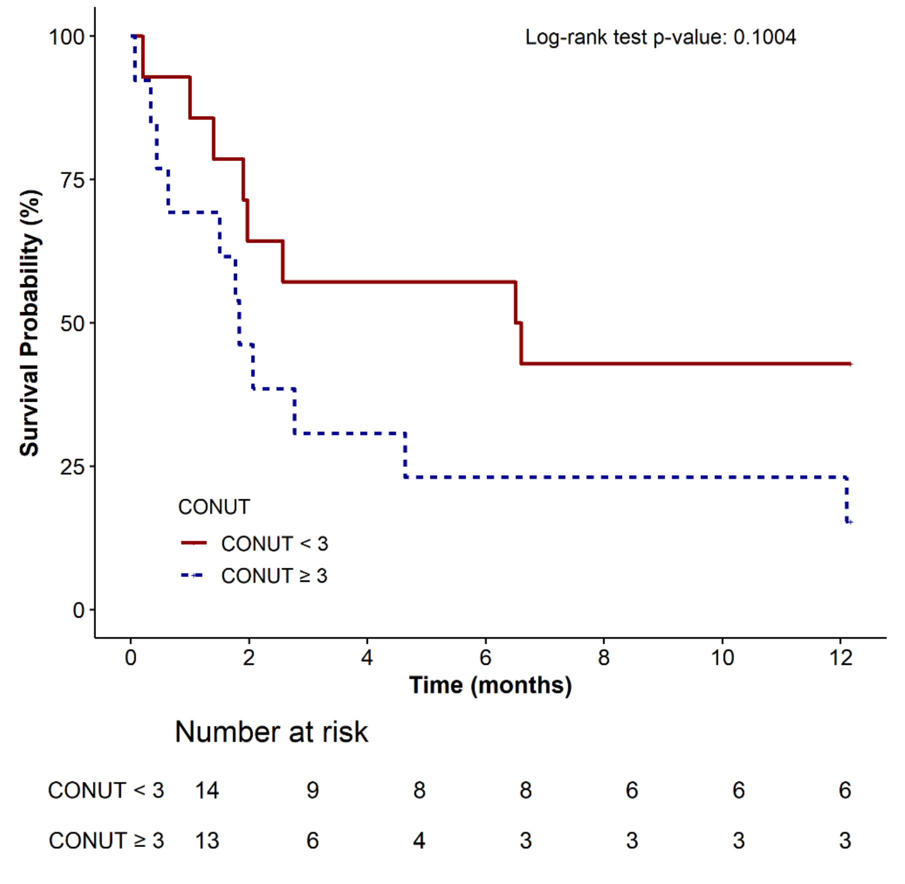

Supplemental Table S2. Cox proportional hazard model of 1-year mortality.

Variable	Univariate		Multivariate model 1		Multivariate model 2		Multivariate model 3		Multivariate model 4	
	HR (95% CI)	*P* value	HR (95% CI)	*P* value	HR (95% CI)	*P* value	HR (95% CI)	*P* value	HR (95% CI)	*P* value
CONUT	1.233 (1.124–1.353)	<0.001								
PNI	0.903 (0.870–0.937)	<0.001								
GNRI	0.956 (0.935–0.976)	<0.001								
TCBI (per 100)	0.991 (0.961–1.021)	0.540								
CONUT										
< 3	ref		ref							
≥ 3	2.387 (1.572–3.624)	<0.001	2.071 (1.345–3.187)	<0.001						
PNI										
> 42	ref				ref					
≤ 42	2.632 (1.662–4.170)	<0.001			1.788 (1.092–2.928)	0.021				
GNRI										
> 102	ref						ref			
≤ 102	2.047 (1.359–3.084)	0.001					1.630 (1.075–2.472)	0.022		
Albumin (g/dL)	0.357 (0.247–0.515)	<0.001							0.436 (0.288–0.660)	<0.001
Male gender	0.829 (0.549–1.252)	0.373								
Age (yr)	1.046 (1.025–1.067)	<0.001	1.026 (1.003–1.050)	0.024	1.021 (0.997–1.046)	0.087	1.032 (1.008–1.056)	0.009	1.023 (1.000–1.047)	0.052
BMI (kg/m^2)	0.961 (0.902–1.023)	0.209								
Tumor size (cm)	1.105 (1.021–1.197)	0.014	1.029 (0.943–1.122)	0.525	1.034 (0.951–1.126)	0.432	1.042(0.956–1.136)	0.348	1.008 (0.927–1.095)	0.858
T stage										
T2	ref									
T3a	0.651 (0.126–3.356)	0.608								
T3b	0.951 (0.311–2.907)	0.930								
T4	1.383 (0.559–3.424)	0.483								
N stage										
N0	ref									
N1	1.930 (1.002–3.720)	0.049								
M stage										
M0	ref									
M1	2.694 (1.621–4.478)	<0.001								
Metastasis										
Lung	2.450 (1.552–3.867)	<0.001								
Bone	1.386 (0.865–2.220)	0.175								
Brain	1.157 (0.631–2.122)	0.637								
Pancreas	1.418 (0.448–4.490)	0.553								
Adrenal gland	2.341 (0.736–7.444)	0.15								
Liver	1.051 (0.386–2.863)	0.923								

Mediastinum	1.951 (0.893–4.263)	0.094								
Staging										
IVa	ref		ref		ref		ref		ref	
IVb	2.733 (0.631–11.830)	0.179	1.596 (0.361–7.053)	0.5374	1.843 (0.418–8.118)	0.419	1.597 (0.360–7.087)	0.538	1.622 (0.368–7.149)	0.523
IVc	6.226 (1.526–25.393)	0.011	3.397 (0.815–14.157)	0.0931	3.365 (0.804–14.084)	0.097	3.404 (0.815–14.219)	0.093	3.100 (0.741–12.971)	0.121
Surgery	0.397 (0.262–0.601)	<0.001	0.593 (0.368–0.955)	0.0315	0.611 (0.380–0.983)	0.043	0.597 (0.370–0.963)	0.035	0.576 (0.356–0.931)	0.024
Surgery type										
Excisional biopsy	ref									
Debulking	0.548 (0.282–1.065)	0.076								
Complete resection	0.402 (0.206–0.784)	0.008								
Chemotherapy	0.842 (0.491–1.444)	0.533								
Target therapy	1.002 (0.668–1.504)	0.991								
Radiation therapy	0.554 (0.334–0.917)	0.022								
Radiation therapy, neck dose (Gy) (per 100)	0.987 (0.979–0.994)	0.001								
Radiation therapy, other site dose (Gy) (per 100)	0.997 (0.968–1.027)	0.848								
Calcium (mg/dL)	1.074 (0.797–1.447)	0.640								
Inorganic Phosphorus (mg/dL)	0.650 (0.478–0.884)	0.006								
Glucose (mg/dL)	1.003 (0.997–1.009)	0.278								
BUN (mg/dL)	1.039 (1.008–1.072)	0.015								
Creatinine (mg/dL)	0.933 (0.521–1.674)	0.817								
Uric acid (mg/dL)	0.892 (0.772–1.030)	0.12								
Total protein (g/dL)	0.996 (0.991–1.001)	0.100								
Total bilirubin (mg/dL)	0.631 (0.467–0.854)	0.003								
Alkaline phosphatase (IU/L)	1.458 (0.667–3.188)	0.344								
Aspartate aminotransferase (IU/L)	1.008 (1.004–1.012)	<0.001								
Alanine aminotransferase (IU/L)	0.968 (0.937–1.000)	0.053								

Triglyceride (mg/dL)	0.972 (0.950–0.994)	0.012
HDL-cholesterol (mg/dL)	1.001 (0.997–1.005)	0.589
LDL-cholesterol (mg/dL)	0.965 (0.934–0.997)	0.035
Calcium (mg/dL)	0.999 (0.987–1.011)	0.864
HbA1c (%)	1.141 (0.713–1.825)	0.584
White blood cell (10^3/μL)	1.047 (1.029–1.066)	<0.001
Hemoglobin (g/dL)	0.800 (0.704–0.909)	0.001
Hematocrit (%)	0.918 (0.876–0.962)	<0.001
Red cell distribution width (%)	1.208 (1.048–1.392)	0.009
Platelet (10^3/μL)	1.001 (0.999–1.003)	0.200
Neutrophil (10^3/μL)	0.863 (0.607–1.226)	0.410
Erythrocyte Sedimentation Rate (mm/hr)	1.052 (1.032–1.072)	<0.001
C-Reactive Protein (mg/L)	1.007 (0.999–1.016)	0.099
eGFR (mL/min/1.73m^2)	1.010 (1.006–1.013)	<0.001
HbA1c (%)	1.004 (0.998–1.011)	0.208

Abbreviations: HR, hazard ratio; CI, confidence interval; CONUT, Controlling Nutritional Status; PNI, Prognostic Nutritional Index; GNRI, Geriatric Nutritional Risk Index; eGFR, estimated glomerular filtration rate; HbA1c, glycated hemoglobin.

Note: Variables were tested separately in multivariate models to avoid collinearity. Continuous and categorical forms were not included in the same model.

Supplemental Table S3. Interaction analysis of pretreatment Controlling Nutritional Status (CONUT) score and each treatment modality (surgical treatment, chemotherapy, targeted therapy, radiation therapy) for predicting 1-year and 2-year mortality in anaplastic thyroid cancer.

Variable	1-year mortality			2-year mortality		
	**HR (95% CI)**	***P* value**	***P* for Interaction (with CONUT ≥ 3)**	**HR (95% CI)**	***P* value**	***P* for Interaction (with CONUT ≥ 3)**
Surgical Treatment			0.080			0.031
CONUT < 3	0.296 (0.088–0.994)	0.049		0.301 (0.090–1.003)	0.051	
CONUT ≥ 3	2.081 (0.267–16.19)	0.484		2.237 (0.289–17.333)	0.441	
Chemotherapy			0.058			0.018
CONUT < 3	1.068 (0.432–2.644)	0.886		1.223 (0.550–2.717)	0.622	
CONUT ≥ 3	0.343 (0.148–0.791)	0.012		0.332 (0.147–0.749)	0.008	
Targeted therapy			0.555			0.231
CONUT < 3	0.673 (0.383–1.181)	0.168		0.805 (0.479–1.354)	0.414	
CONUT ≥ 3	0.654 (0.304–1.405)	0.277		0.616 (0.293–1.297)	0.203	
Radiation therapy			0.819			0.636
CONUT < 3	0.473 (0.217–1.033)	0.060		0.589 (0.282–1.230)	0.158	
CONUT ≥ 3	0.376 (0.168–0.843)	0.018		0.383 (0.176–0.835)	0.016	

Values represent hazard ratios (HRs) with 95% confidence intervals (CIs).

Multivariable Cox proportional hazards models were used to assess the interaction between pretreatment CONUT score (<3 vs. ≥3) and each treatment modality (Surgery, Chemotherapy, Targeted Therapy, Radiation Therapy). Hazard ratios (HRs) represent the effect of each treatment (yes vs. no) stratified by CONUT group. All models were adjusted for age, tumor size, TNM stage, and surgery (except when surgery was the treatment variable).

Abbreviations: CONUT, Controlling Nutritional Status.
