# Supplementary figures and images for: Prognostic Impact of the Pretreatment Controlling Nutritional Status (CONUT) Score in Anaplastic Thyroid Cancer: A Retrospective Cohort Study
Source: Cancers (Basel). 2025 Oct 16;17(20):3344. doi: 10.3390/cancers17203344 (PMC12564293; doi:10.3390/cancers17203344)

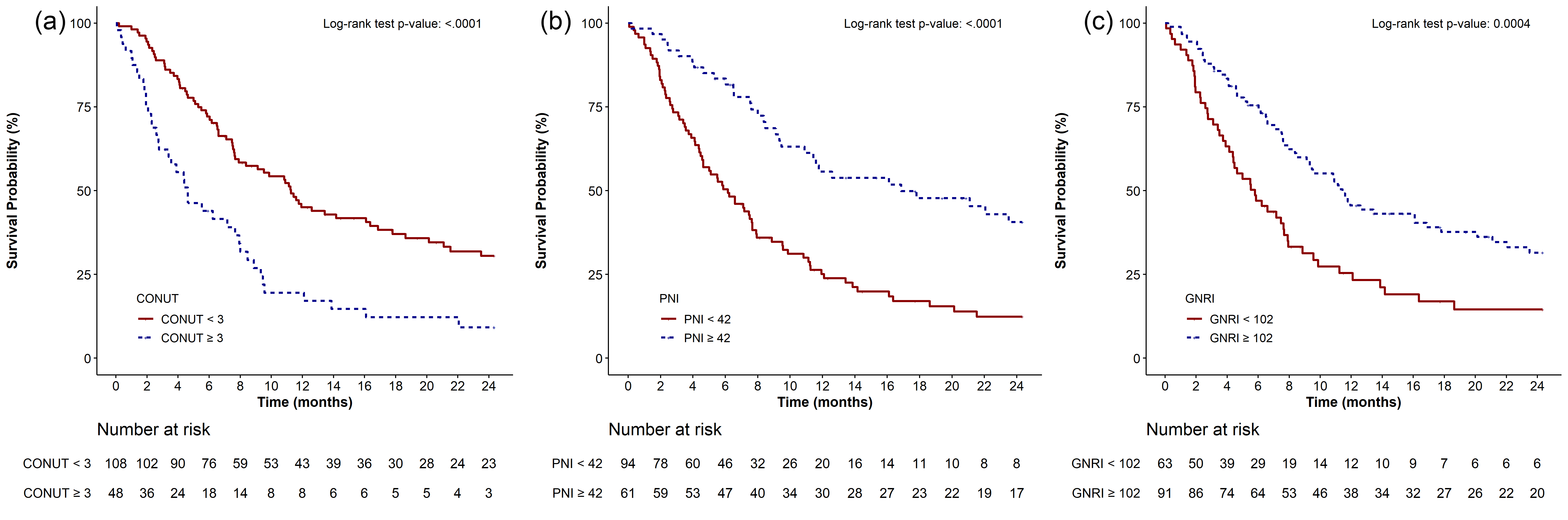

Supplement: Supplementary file 1 [file cancers-17-03344-s001.zip › Supplementary Figure S1. KM curves 2-year.tif]

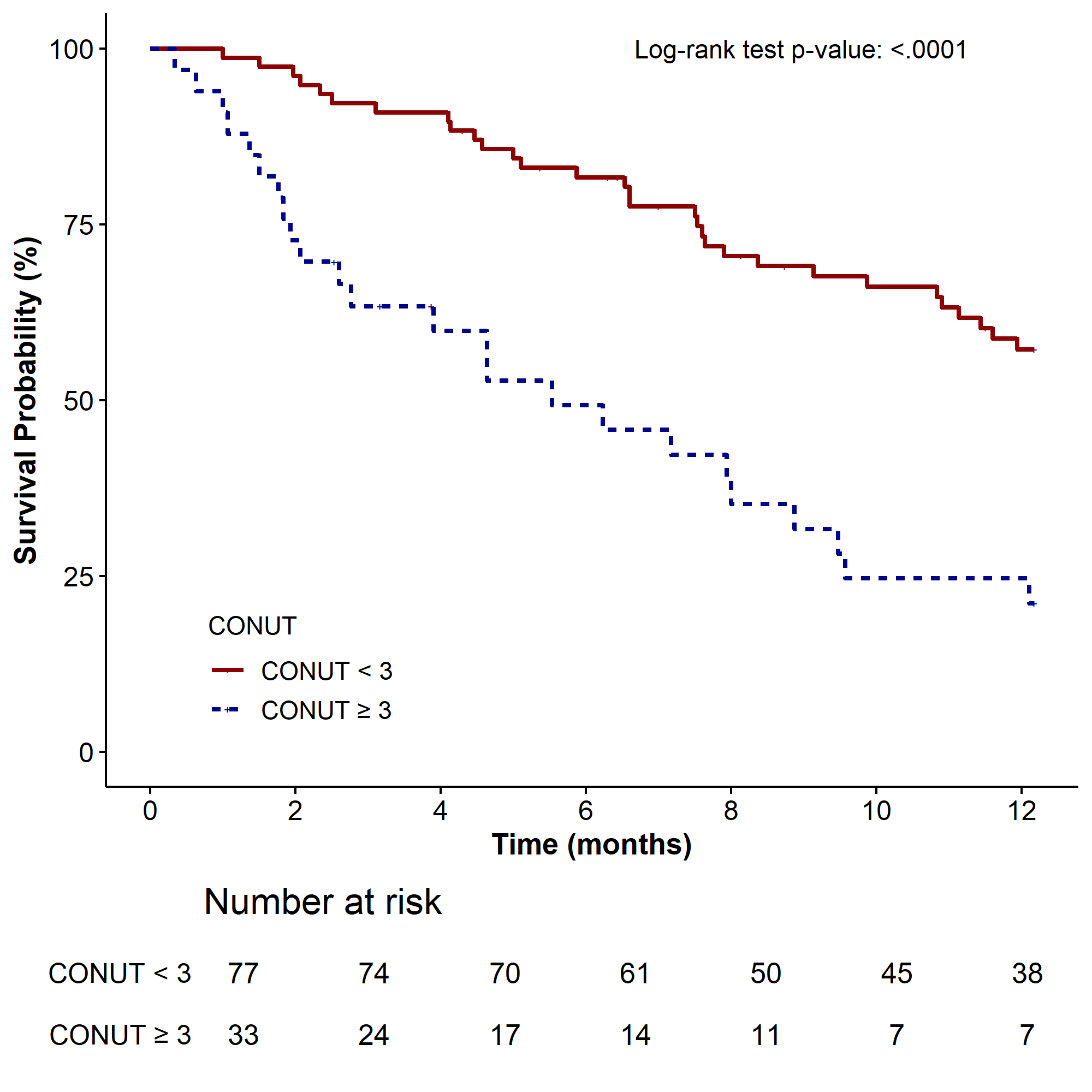

Supplement: Supplementary file 1 [file cancers-17-03344-s001.zip › Supplementary Figure S2. a.tiff]

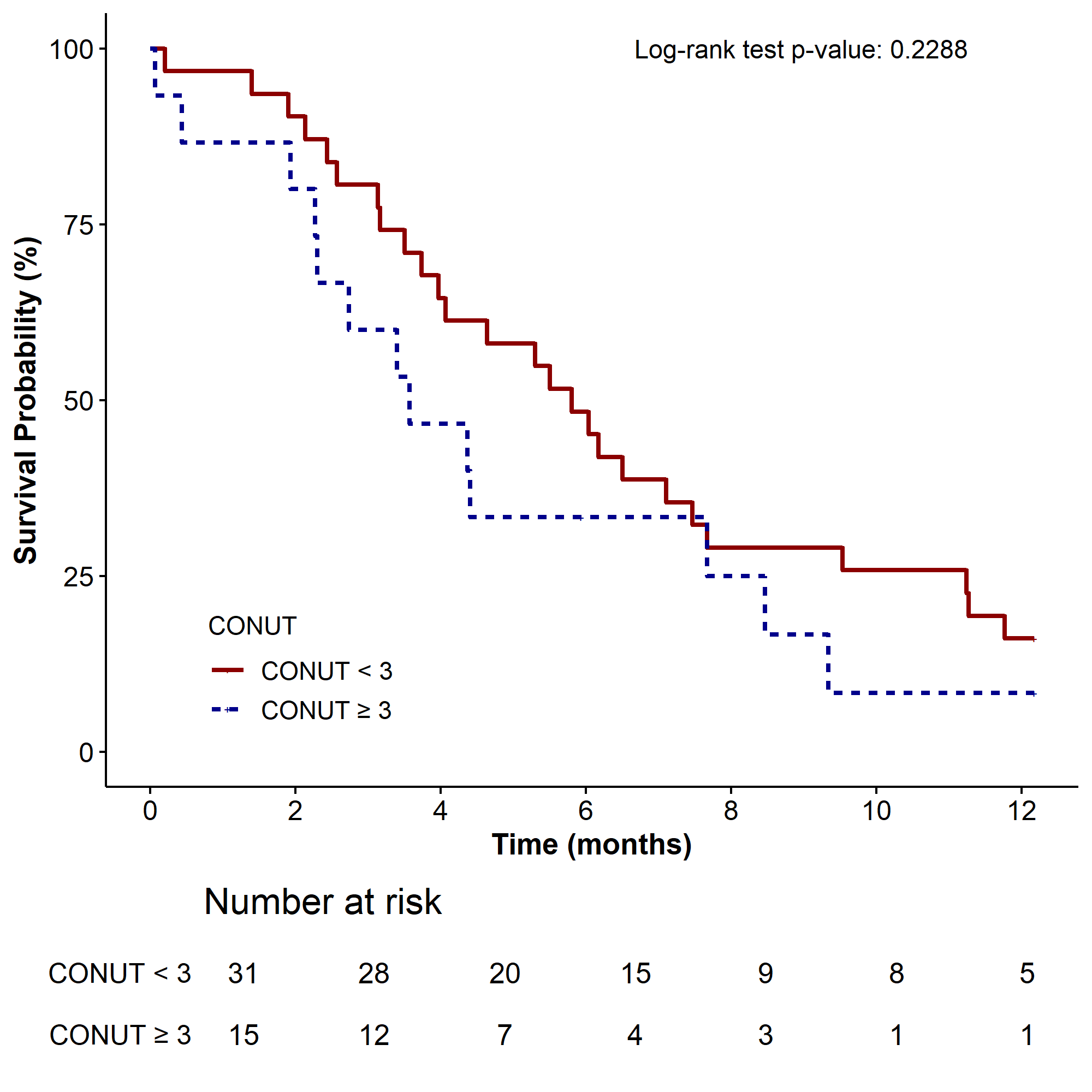

Supplement: Supplementary file 1 [file cancers-17-03344-s001.zip › Supplementary Figure S2.b.tiff]

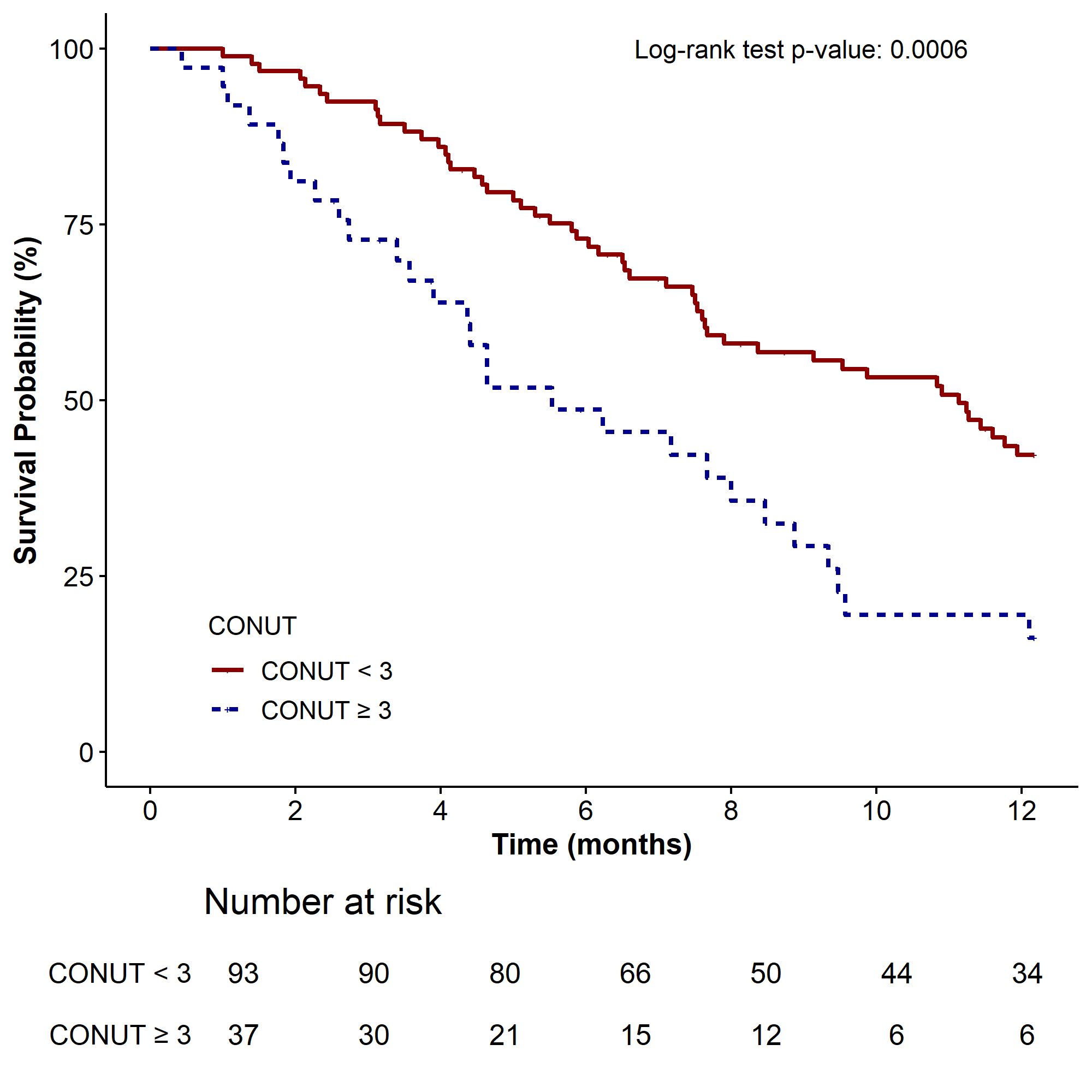

Supplement: Supplementary file 1 [file cancers-17-03344-s001.zip › Supplementary Figure S3. a.tiff]

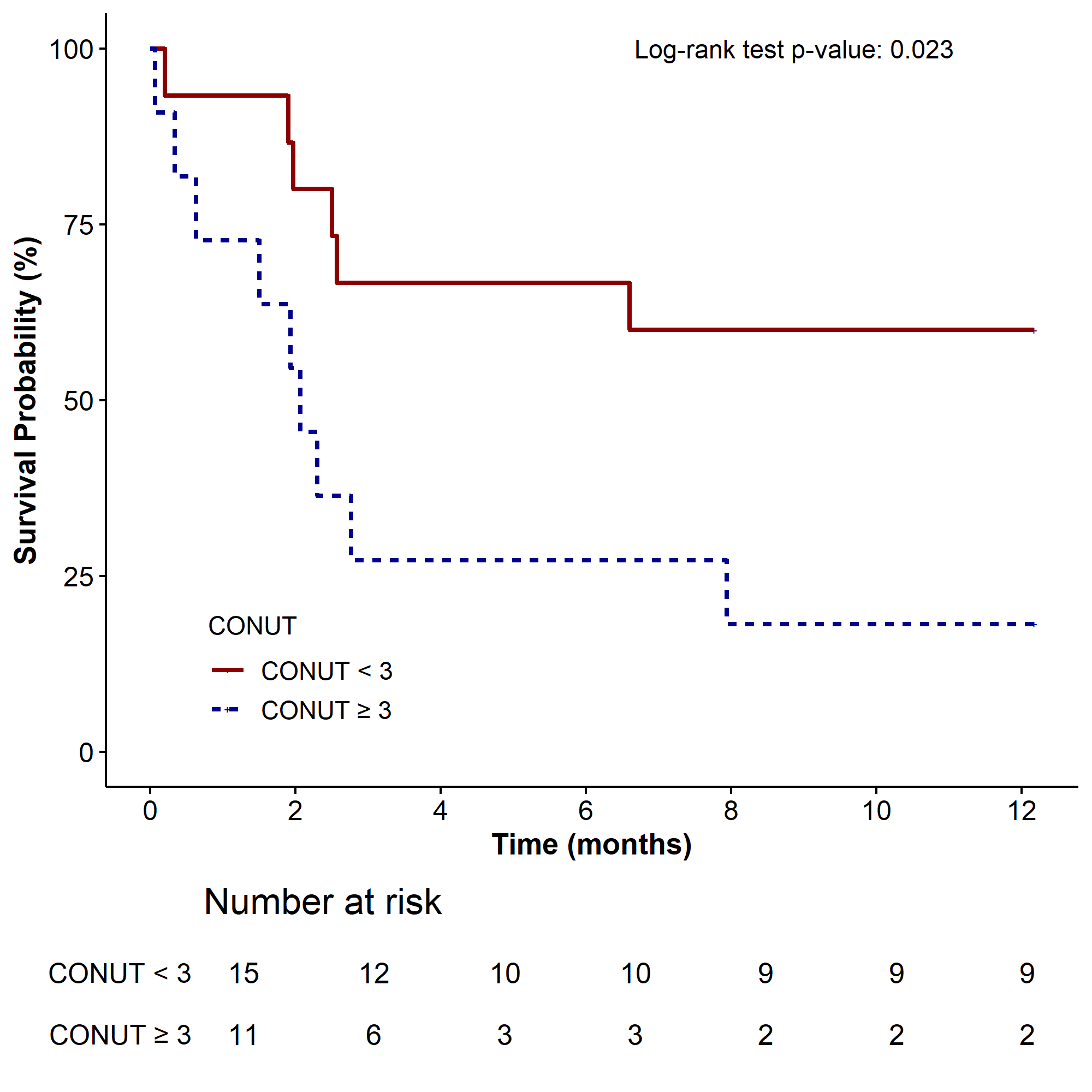

Supplement: Supplementary file 1 [file cancers-17-03344-s001.zip › Supplementary Figure S3. b.tiff]

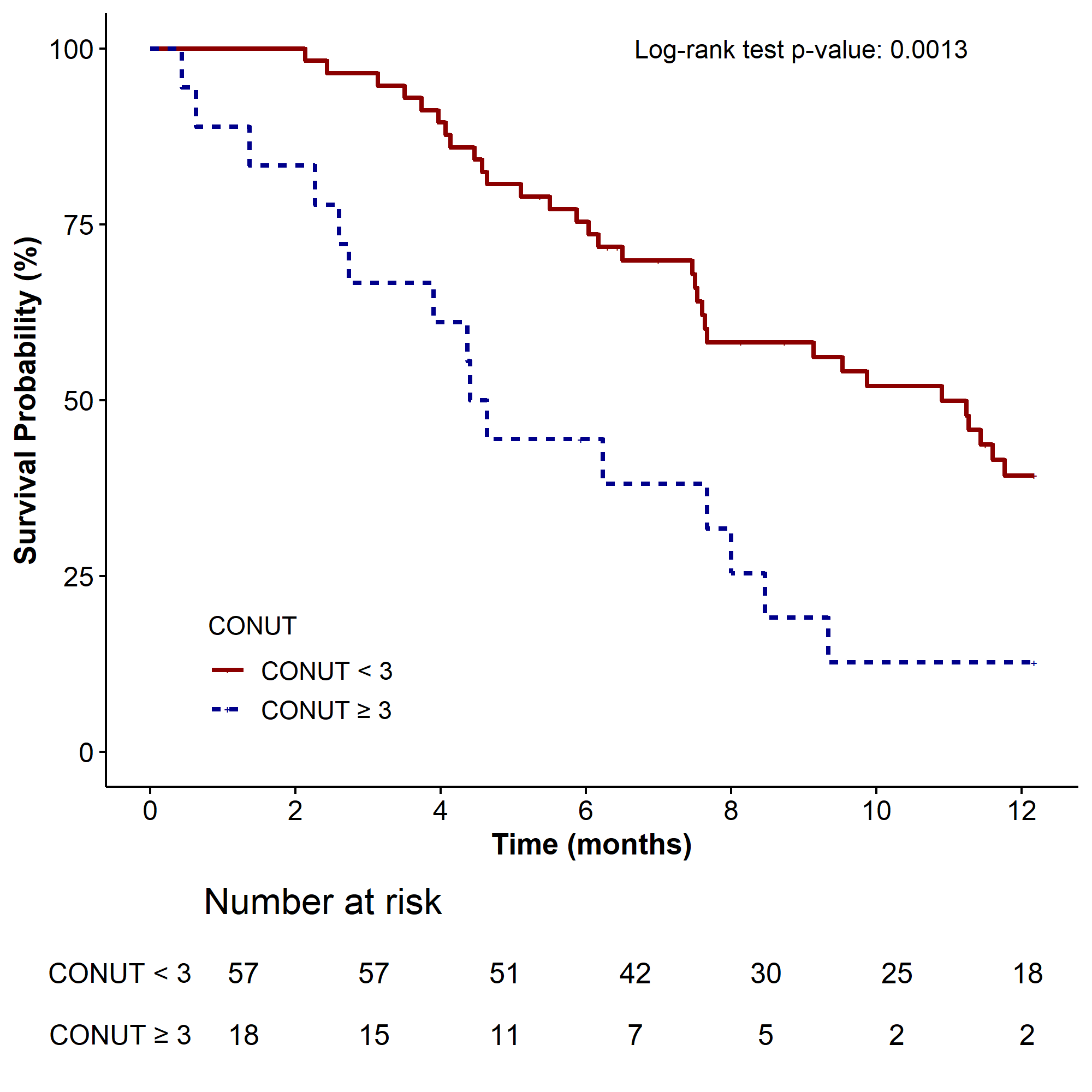

Supplement: Supplementary file 1 [file cancers-17-03344-s001.zip › Supplementary Figure S4. a.tiff]

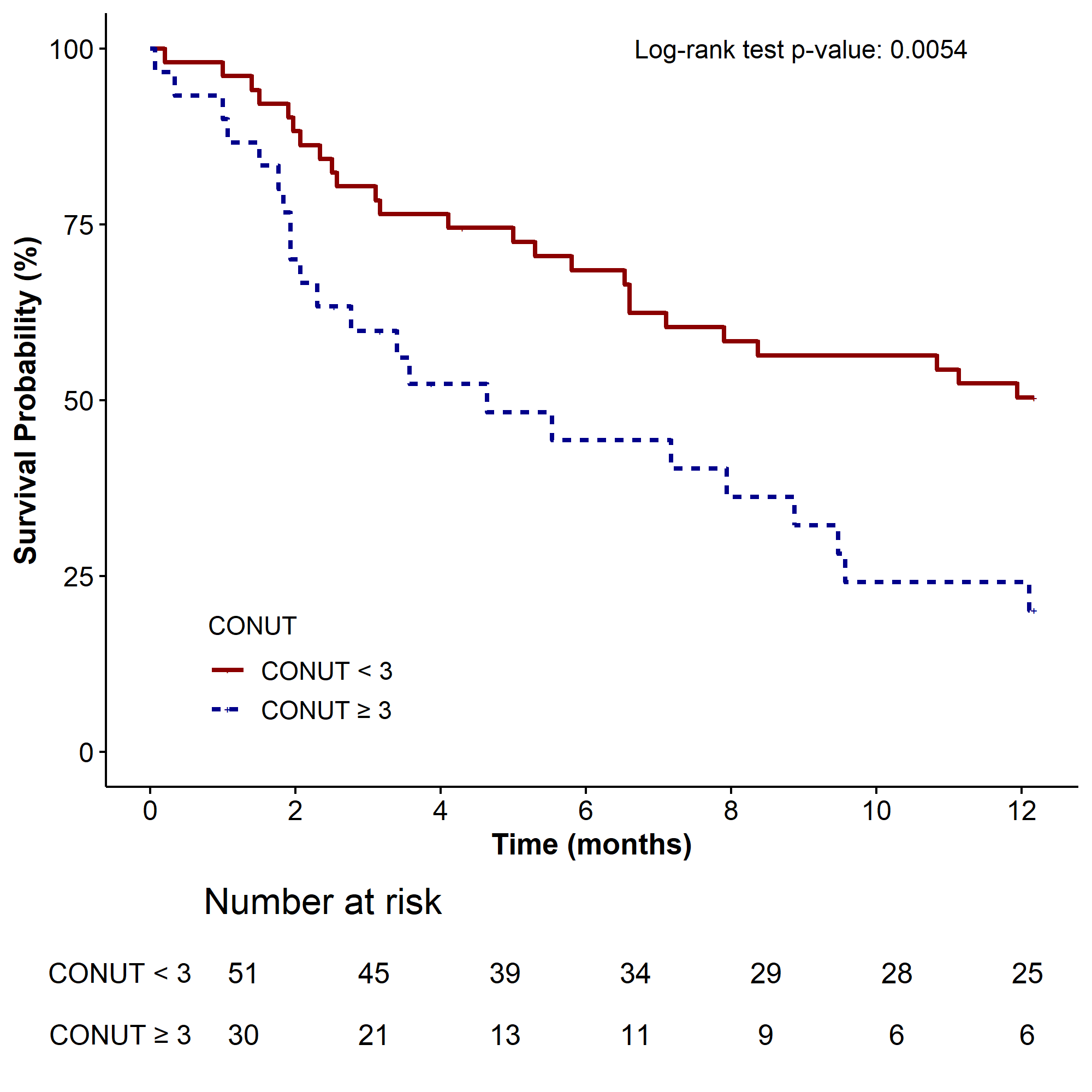

Supplement: Supplementary file 1 [file cancers-17-03344-s001.zip › Supplementary Figure S4. b.tiff]

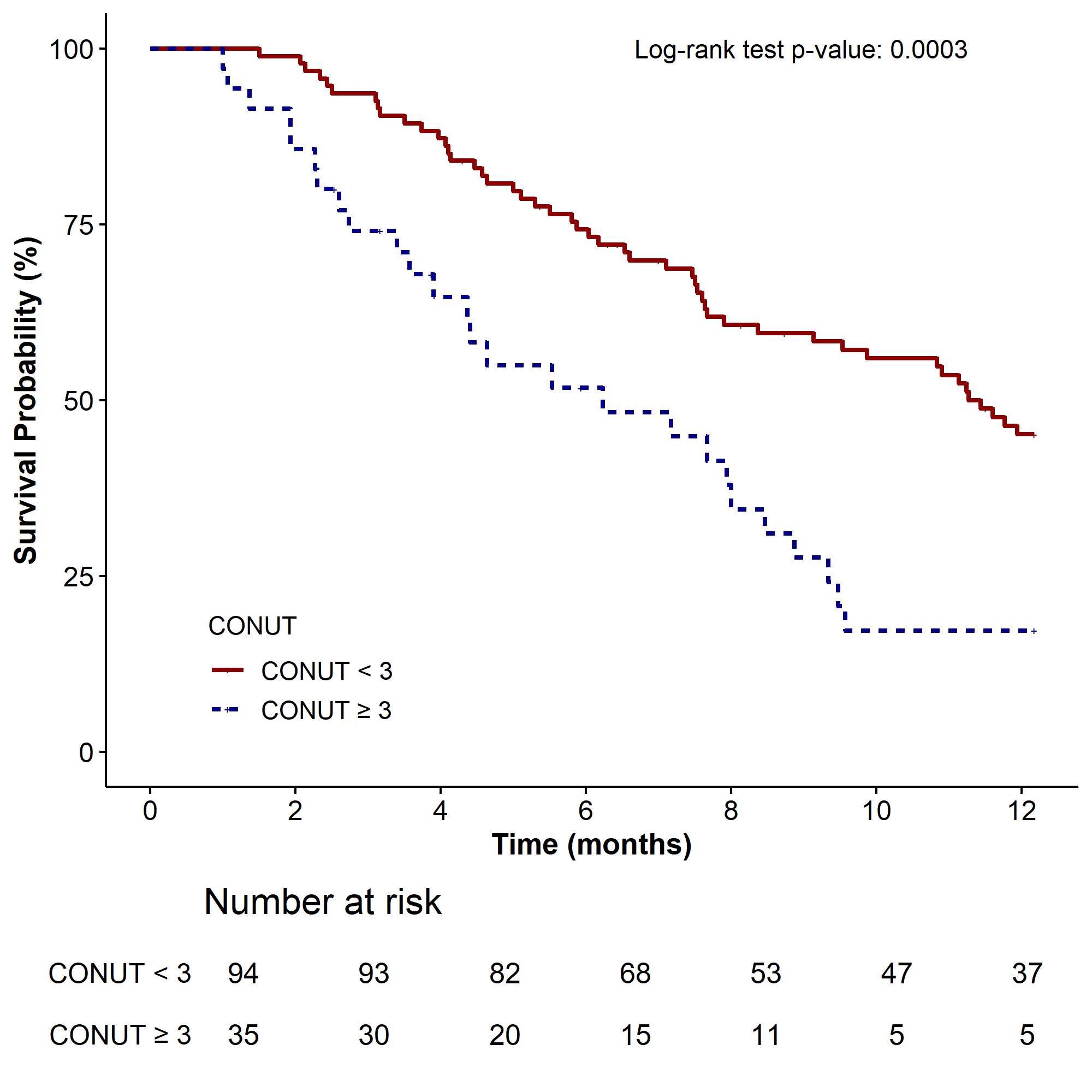

Supplement: Supplementary file 1 [file cancers-17-03344-s001.zip › Supplementary Figure S5. a.tiff]

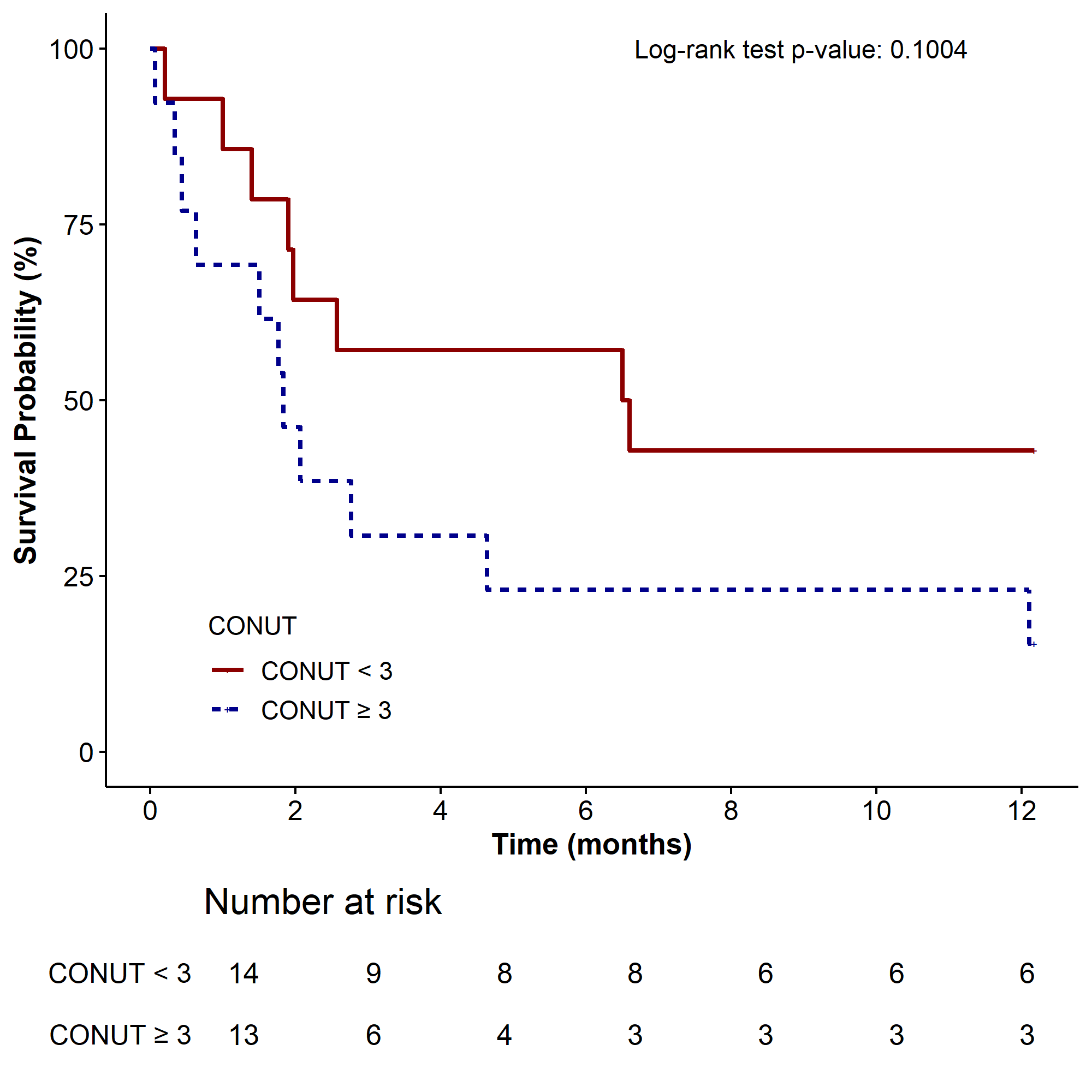

Supplement: Supplementary file 1 [file cancers-17-03344-s001.zip › Supplementary Figure S5. b.tiff]
